# Up-regulation of hypoxia-inducible factor antisense as a novel approach to treat ovarian cancer

**DOI:** 10.7150/thno.41792

**Published:** 2020-05-25

**Authors:** Tiangong Lu, Jianming Tang, Binita Shrestha, Blake R. Heath, Li Hong, Yu L. Lei, Mats Ljungman, Nouri Neamati

**Affiliations:** 1 Department of Medicinal Chemistry, College of Pharmacy, Rogel Cancer Center, University of Michigan, Ann Arbor, MI 48109-2800, USA; 2 Department of Gynecology and Obstetrics, Renmin Hospital of Wuhan University, 238 Jiefang Road, Wuhan, Hubei province, 430060, P.R. China; 3 Departments of Periodontics and Oral Medicine and Otolaryngology- Head and Neck Surgery, Rogel Cancer Center, University of Michigan, Ann Arbor, MI 48109-2800, USA; 4 Department of Radiation Oncology, University of Michigan Medical School, Rogel Cancer Center and Department of Environmental Health Sciences, School of Public Health, University of Michigan, Ann Arbor, MI 48109-2800, USA

**Keywords:** SC144, hypoxia, antisense RNA, iron chelators, ovarian cancer

## Abstract

Ovarian cancer (OC) is estimated to kill ~14,000 women in the United States in 2019. Current chemotherapies to treat OC initially show therapeutic efficacy but frequently drug resistance develops, at which point therapies with alternative targets are needed. Herein, we are describing a novel approach to sensitize these tumors to standard chemotherapies by increasing the transcription of hypoxia-inducible factor antisense.

**Methods:** Genome-wide Bru-seq analysis was performed to fully capture the nascent transcriptional signature of OC cells treated with the gp130 inhibitor, SC144. *In vitro* and *in vivo* analysis, including characterization of hypoxia and select protein expression, combination with standard of care chemotherapy and antitumor efficacy were performed to assess the biological activity of SC144 on induction of hypoxia in OC cells.

**Results:** Bru-seq analysis of OVCAR8 cells treated with SC144 shows upregulation of hypoxia related genes. In addition, transcription of hypoxia-inducible factor antisense (HIF1A-AS2) was induced that in turn reduced expression of HIF-1α and simultaneously increased expression of NDRG1. Furthermore, we observed decreased protein levels of EGFR, Met, c-Myc, cyclin D1, MMP-2, MMP-9 and TF, and phosphorylation of Src and P130-cas. SC144-induced alterations of HIF-1α and NDRG1 were also confirmed in prostate cancer cells. Ciclopirox olamine (CPX) induces a cellular transcriptional profile comparable to SC144, suggesting a similar cellular mechanism of action between these two compounds. In addition, SC144 sensitized OC cells to olaparib, carboplatin and cisplatin, and shows better *in vivo* efficacy than CPX.

**Conclusion:** Induction of hypoxic stress responses through inhibition of gp130 represents a novel approach to design effective anticancer treatments in combination with standard-of-care chemotherapy in OC and the efficacy reported here strongly supports their clinical development.

## Introduction

Ovarian cancer (OC) is the fifth-leading cause of death in women and ranks first in gynecologic cancer deaths in the United States 1. Due to the lack of early symptoms and effective screening strategies, OC is usually diagnosed at an advanced stage 1, 2. Current chemotherapeutic drugs for the treatment of OC include carboplatin and paclitaxel. In addition, three poly(ADP-ribose) polymerase (PARP) inhibitors, olaparib, niraparib, and rucaparib, have been approved as a monotherapy or following platinum chemotherapy as maintenance therapy 3-5. The efficacy of combination chemotherapy is considered superior to monotherapy for OC treatment. However, due to drug resistance and drug-induced side effects, the efficacy of first-line chemotherapeutic drugs has been limited. Therefore, developing new drugs with improved safety and efficacy are urgently needed for the treatment of OC. Therefore, compounds that can induce chronic depletion of HIF-1α protein and exacerbate hypoxia damage can be uniquely used to treat various cancers including OC.

Hypoxia is well-documented to occur during tumorigenesis as tumors grow and become less oxygenated. Under hypoxic stress conditions, normal cells undergo cellular senescence, mitotic arrest, and programmed cell death with damaged DNA 6-8. Cancer cells survive under hypoxia via the upregulation of hypoxia-inducible factors (HIFs). HIFs in turn activate a series of pathways regulating proliferation, angiogenesis, and invasion, while inhibiting apoptosis-related factors and pathways 9-11. Upregulation of HIF-1α mRNA and protein levels has been reported to be involved in OC resistance to therapy and development of metastasis both *in vitro* and *in vivo* 2, 7, 12. Down-regulation of HIFs or its downstream factors in cancers promotes cell death and therefore represents a promising target. A variety of HIF inhibitors have been developed for cancer therapy: for example, PX-748, 2ME2, TAT-cyclo-CLLFVY are HIF-1α inhibitors 13-15, and TC-S 7009 is HIF-2α inhibitors 16. However, low *in vivo* efficacy or high *in vivo* toxicity has limited their clinical development 11, 17, 18.

Non-coding RNAs (ncRNAs) that constitute the majority of the human transcriptome have been increasingly recognized as important regulators of gene expression. Long non-coding RNA (lncRNA) are involved in physiological processes, such as gene transcription and post-translational regulation, and have been functionally associated with many human diseases, including cancers 19, 20. A large number of studies have shown that lncRNAs are either positively or negatively correlated with hypoxia-related tumor progression and metastasis, suggesting that lncRNAs can serve as diagnostic and prognostic biomarkers in many types of cancers. As an example, differentiation antagonizing non-protein coding RNA (DANCR) increases HIF-1α mRNA stability and promotes nasopharyngeal carcinoma (NPC) cell invasion and metastasis 21. Metastasis-associated protein 2 (MTA2) stabilizes the HIF-1α protein via deacetylation in pancreatic cancer, and its transcriptional regulator RNA MTA2TR is transcriptionally regulated by HIF-1α under hypoxic conditions 22. Conversely, lncRNA- CF129 indirectly inhibits HIF-1α expression via lncRNA-interacted feedback of HIF-1α and FOXC2, and reduced in pancreatic cancer during hypoxia microenvironment 23.

Hypoxia-inducible factor 1 alpha-antisense RNA 2 (HIF1A-AS2, aHIF) is induced by and negatively regulates HIF-1α under hypoxic conditions. HIF-1α protein is stabilized and accumulates in response to acute hypoxia (4 h, 0.5% O_2_). During sustained hypoxia (12 h, 0.5% O_2_), HIF-1α proteins increase HIF1A-AS2 transcript that in turn triggers HIF-1α mRNA decay and eventually decreases HIF-1α protein expression 24. HIF1A-AS2 transcript has been detected in many human tissues, and has been linked with poor prognosis in various cancers, including breast 25, kidney 26 and gastric 27. In a study of antisense loci dysregulation in lung adenocarcinoma and normal samples, tumor as well as matched normal tissues expressed HIF1A sense/ antisense in a concordant manner, which means sense and antisense are either both overexpressed or under expressed 28. HIF1A-AS2 drives tumor progression and is considered to be a non-protein coding oncogene in mesenchymal GBM stem-like cells (M-GSCs) but not in proneural GBM stem-like cells (P-GSCs) 20. shRNA-mediated knockdown of HIF1A-AS2 in M-GSCs resulted in significant decrease in cell growth and viability *in vitro*, smaller tumors, and prolonged survival in mice. Additionally, HIF1A-AS2 could enhance the expression of several mRNA targets, such as HMGA1 (high mobility group AT-hook 1) mRNA, and subsequent molecular response to hypoxic stress through the direct interaction with IGF2BP2 (insulin-like growth factor 2 mRNA binding protein 2) and DHX9 (ATP-dependent RNA helicase A) 20, 29. There is a growing interest in better elucidating the role of non-coding RNA in controlling iron metabolism 30, 31.

Iron is an essential element in cellular metabolism and growth, such as energy production, DNA synthesis and oxygen transport. Due to the increased iron requirements, cancer cells are far more sensitive than normal cells to iron depletion 32. Iron chelators are used to treat iron overload disease and have become an effective therapeutic option for cancer treatment 33-35. Iron chelators, such as desferoxamine (DFO), ciclopirox (CPX) and deferasirox (DFX), have been reported to mimic hypoxic induction of HIF-1α in normoxic cells 36. HIF-1α induction by DFO, CPX or DFX, suggests a direct link between iron chelation and hypoxic induction 37. Due to the low *in vivo* activity and/or high *in vivo* toxicity, iron chelators' development as anticancer drugs has been limited 38-41.

Previously, we identified and reported on a novel orally active small-molecule gp130 inhibitor, SC144, that delays tumor growth in a mouse xenograft model of human OC without significant toxicity to normal tissues 42. Herein, we unveil that SC144 inhibits hypoxic response to produce exacerbated hypoxia damage in OC cells. SC144 upregulates several key hypoxic stress mediators, including HK2, PDK1/3, BNIP3, EGLN1, and HMOX-1. SC144-treated cells upregulated HIF1A-AS2 transcription, resulting in modulations of HIF-1α and its downstream gene NDRG1. Notably, a comparable transcription profile was observed with OC cells treated with CPX, suggesting a similar cellular mechanism of action between these two compounds. In addition, SC144 sensitizes OC cells to olaparib, carboplatin and cisplatin in both 2D and 3D cultures and shows better *in vivo* anticancer efficacy than CPX.

## Experimental Procedures

### Reagents

SC144 was synthesized as previously described 43. Olaparib was purchased from Selleckchem. Carboplatin, cisplatin and DFO were purchased from Sigma-Aldrich. Stock solutions of 10 mmol/L SC144 and olaparib were prepared in dimethyl sulfoxide (DMSO) and stored at -20℃. Stock solutions of 10 mmol/L carboplatin and cisplatin were prepared in phosphate-buffered saline (PBS) and stored at -20 ℃. Further dilutions were made fresh in cell culture medium. Anti-GAPDH (2118S), anti-HK2 (2106S), anti-BNIP3 (sc56167), anti-HO-1 (5061S), anti-HIF-1α (3716S), anti-NDRG1 (5196S), anti-EGFR (2232S), anti-Met (8198P), anti-phospho-Src (Y416, 2101S), anti-non-phospho-Src (2107), anti-cyclin D1 (2978), anti-MMP2 (4022), anti-MMP9 (3852), anti-phospho-p130 Cas (Tyr410, 4011S), anti-c-MYC (5065S), anti-cleaved-caspase-3 (9664S), anti-cleaved-PARP (5625S) were purchased from Cell Signaling Technology. Anti-PDK1 (sc293160), anti-PDK3 (sc365378), anti-PHD2 (sc271835), anti-TF (sc-393657) were purchased from Santa Cruz Biotechnology. Hypoxia detection kit (ENZ-51042-0125) was obtained from Enzo Life Sciences, Inc. Trypan Blue 0.4% solution was purchased from Lonza Inc.

### Cell culture

OVCAR-8, SK-OV-3 and LN-Cap cells (National Cancer Institute, Developmental Therapeutics Program, Bethesda, MD) were cultured in RPMI-1640 with 10% heat-inactivated FBS (Gemini-Bioproducts). The HPV16 E6/E7-expressing tumor cell model MOC2-E6/E7 is syngeneic to C57BL/6, and used as previously described 44-46. In brief, cells were cultured in Iscove's modified Dulbecco's medium/ F12 (2:1) with 5% FBS, penicillin/streptomycin, 5 ng/mL EGF (Millipore), 40 ng/mL hydrocortisone, 5 mg/mL insulin, and 4 mg/mL puromycin. All experiments were carried out using cells in the exponential growth phase. Cells were routinely checked for mycoplasma contamination by using PlasmoTest (InvivoGen).

### Bru-seq analysis of nascent RNA synthesis

Bru-seq analysis was performed as previously reported 47, 48. Briefly, 4×10^6^ OVCAR-8 and LNCaP cells in the logarithmic growth phase were seeded in 10 cm dishes. When cells reached 80-90% confluence, cells were either mock-treated or treated with drugs at indicated times (4h or 24h). Finally, bromouridine (2 mM) was added to the media during the last 30 min of treatment to label newly synthesized nascent RNA. Cells were lysed in TRIzol (Invitrogen) and total RNA was isolated. Bru-labeled, nascent RNA was isolated using anti-BrdU antibodies (BD Biosciences, San Jose, CA, USA) conjugated to magnetic beads (Invitrogen). The isolated RNA was converted into cDNA libraries and sequenced at the University of Michigan Sequencing Core using an Illumina HiSeq 2000 sequencer (San Diego, CA). Sequencing reads were mapped to hg38 reference sequence and analyzed as previously described 47, 48.

Only measurements mapping to Entrez identifiers were considered and genes were considered significantly differentially expressed with absolute fold change > 2 and mean RPKM > 0.5. Gene set enrichment of differentially expressed genes was performed using DAVID Bioinformatics Resource 6.7 (https://david.ncifcrf.gov/), gene set enrichment analysis v2.2.3 (GSEA, Broad Institute, MA) and Connectivity Map L1000 Platform (CMap) 49-51, with a background of all measured protein coding genes. Gene sets with false discovery rate (FDR) *q*-values < 0.25 were considered to be significantly enriched and were used in the analysis.

### MTT assay

Growth inhibition in OVCAR-8 and SK-OV-3 cells (3000 cells/well) was assessed using MTT assay as described previously 42. All assays were conducted in triplicate. Percentage of cell growth inhibition was expressed as: (1-A/C) × 100% (A and C were the absorbance values from experimental and control cells, respectively). The synergistic effect was evaluated using Combenefit software (v2.021) and HSA (Highest Single Agent) analyses 52.

### Western blotting

After treatments, OVCAR-8 and SK-OV-3 cells (only attached cells were collected) were washed with ice-cold PBS and lysed in cold lysis buffer (20 mmol/L Tris-HCl, 150 mmol/L NaCl, 1 mmol/L EDTA, 1% Triton X-100, pH 7.5) with protease and phosphatase inhibitors. Total protein concentration was determined by BCA kit (Thermo Scientific) and Western blotting was conducted as described 42. Detailed information, dilutions and validations of antibodies are provided in the Supplementary Section.

### Colony formation assay

Colony formation assays in OVCAR-8 and SK-OV-3 cells (300 cells/ well) were conducted as described 53. The colonies were imaged using iBright Imaging Systems (Thermo Fisher Scientific, FL1000).

### Hypoxia assay

Nitroreductase activity in hypoxic cells was measured using a Hypoxia detection kit (ENZ-51042-0125) according to the manufacturer's instructions. Red Hypoxia Detection Reagent (probe) is a nonfluorescent aromatic compound containing a nitro moiety, which could be converted to hydroxylamine (NHOH) and amino (NH2) group by the nitroreductase presented in hypoxic cells, subsequently releasing the fluorescent probe. Briefly, cells were seeded at the density of 3000 cells/ well in 4-chamber culture slides (Falcon, 35104). After overnight attachment, cells were treated with SC144 in fresh media at a final concentration of 1.2 μM for various time points (2, 6, 12, 24 and 48 h). DFO (200 μM for 4 h) was used as positive control. Cells were washed twice with PBS, and incubated with 500 μL of hypoxia detection mix (5 µL hypoxia detection reagent in 10 mL culture medium) for 30 min. Next, hypoxia detection mix was removed, and cells were washed twice with PBS. Cells were imaged by a fluorescence microscope (Olympus Corporation) using standard excitation/emission filter sets (Ex/Em: 596/670 nm).

### Scratch assay

Cells were seeded at the density of 5×10^5^ cells/well in 6-well plate in RPMI 1640 with 10% FBS and scratch assay was performed as described 54. Cells were washed with PBS for two times, different concentrations of SC144 in RPMI 1640 with 1% FBS were added and incubated for 24 h. Cells were stained with crystal violet solution (2%) for 30 min and thoroughly washed with water. Stained cells were imaged on a microscope (Olympus Corporation) using a 10× objective. Data presented is a representative image of at least three independent experiments.

### 3D spheroid assay

Cells were seeded in 96 well clear black round bottom ultra-low attachment spheroid microplate (Corning®, 4515), at 3000 cells/well with 0.015 mg/well collagen. The next day, single or multiple viable spheroids were generated and treated with SC144, olaparib, caboplatin and cisplatin at different concentrations. On Day 7, the spheroids were imaged using a microscope. Cell viability was measured with the CellTiter-Glo® 3D Cell Viability Assay (Promega, Cat. #G9682), following the manufacturer's instructions. Luminescence was measured at room temperature using a Synergy H1 Hybrid Multi-Mode Microplate Reader (BioTek Instruments, Inc). The synergistic effect was evaluated using Combenefit software (v2.021) with the HSA model.

### Mice

Mice were fed ad libitum and kept in air-conditioned rooms at 20℃±2℃ with a 12-hour light-dark period. Animal care and manipulation were in agreement with the University of Michigan institutional guidelines, which were in accordance with the Guidelines for the Care and Use of Laboratory Animals.

### *In vivo* tumor xenograft studies

MOC2-E6/E7cells were implanted subcutaneously on the flank of 6- to 8-week-old C57BL/6 mice (Jackson Laboratory). PBS (control) or SC144 (40 mg/kg) were injected intraperitoneally daily for two weeks. CT-26 cells were implanted subcutaneously into the dorsal flanks of C57BL/6 mice (1 × 10^6^ cells in 100 mL of PBS/mouse) under aseptic conditions as described 42. Tumor growth was assessed by daily measurement of tumor diameters with a Vernier caliper. Tumor volume was calculated according to the formula: Tumor volume (mm^3^) = D×d^2^/2, where D and d are the longest and shortest diameters, respectively. For intraperitoneal (i.p.) administration, tumors were allowed to grow to an average volume of 80 mm^3^. Mice were then randomly grouped (n = 5 per group). On day 10 to 11, the treatments were conducted daily with SC144 (10 mg/kg) and CPX (10 mg/kg). On day 12 to 14, the treatments were conducted daily with SC144 (40 mg/kg) and CPX (15 mg/kg). On day 15 to 21, the treatments were conducted twice a day with SC144 (40 mg/kg) and CPX (15 mg/kg). The SC144 solution for i.p. administration was containing 10% DMSO, 60% propylene glycol and 30% saline with 0.9% NaCl. The CPX solution for i.p. administration contained 5% DMSO, 50% propylene glycol and 35% saline with 0.9% NaCl. The vehicle solution for i.p. administration contained 10% DMSO, 60% propylene glycol and 30% saline with 0.9% NaCl. The solutions for treatments were prepared as described 42. Mouse body weight and tumor volume were measured twice a week.

### Quantitation of gene expression

Whole tumors were homogenized, and RNA extracted using Qiagen RNeasy Mini Kit (Cat. #74104). Gene expression qPCR primers are: Ifnb1 F 5'-CCAGCTCCAAGAAAGGACGA, R 5'-CGCCCTGTAGGTGAGGTTGAT; Tnf F 5'-ATGAGAAGTTCCCAAATGGC, R 5'-CTCCACTTGGTGGTTTGCTA; Arg1 F 5'- CAGAAGAATGGAAGAGTCAG, R 5'- CAGATATGCAGGGAGTCACC.

### Statistical analysis

Data are expressed as mean ± SD of at least 3 independent experiments. Student's t-test and ANOVA were used for statistical analysis and the p values were determined using SPSS 16.0 (SPSS Inc.) and Prism 7 (GraphPad Software, Inc.). Differences were considered statistically significant at p < 0.05.

## Results

### SC144 induces a hypoxic stress response in ovarian cancer cells

To better understand the potential mechanism of SC144, we first subjected SC144 treated OVCAR-8 cells to Bru-seq analysis to examine the global changes in transcription 47, 48. A total of 1799 genes and gene products (1321 protein-coding and 478 non-coding) were identified as significantly changed by SC144 (Tables S1-4). Homeobox A13 (HOXA13) gene which is upregulated almost 500-fold is reported to be an unfavorable prognostic factor and novel oncogene for prostate cancer 55 and is also suggested to be used as biomarker for prognosis in bladder cancer 56. It is also responsible for gastric cancer progression 57. Another highly up-regulated gene heme oxygenase1 (HMOX1, 68-fold)) has a key role in protecting tumor cells from apoptosis and it is also suggested as a potential marker for prediction of OC prognosis and is a therapeutic target for OC and gastric cancer 58, 59. However, it is down-regulated in inflammatory bowel disease 60. Most of the top 20 upregulated genes in response to SC144 treatment are important for cancer progression, such as PIK3IP1 (37-fold), which suppresses the development of hepatocellular carcinoma and also has inhibitory role in T-cell activation 61, 62. Histone family members, which are responsible for the nucleosome structures of the chromosomal fiber in eukaryotes, compose the most down-regulated genes. Among the top 20 up- and down-regulated non-protein coding genes, genes such as NMRAQL2P, LUCAT 1 and HIF1A-AS2 are associated with various types of cancers. Most of the non-coding genes are not yet reported to have a connection with cancer.

**Figure 1:**
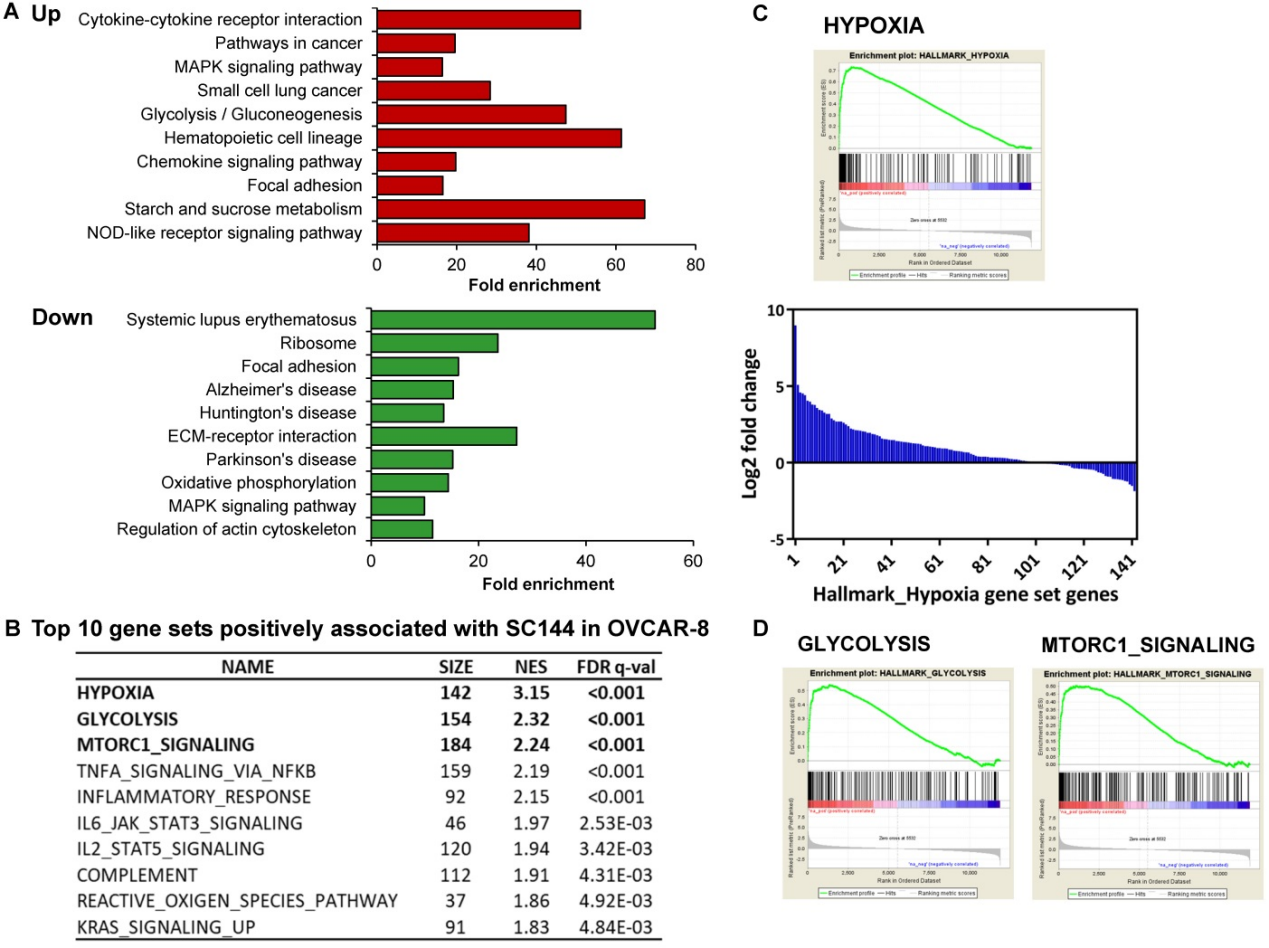
SC144 induces hypoxia signaling. **(A)** KEGG pathway gene enrichment of upregulated and downregulated genes in Bru-seq analyzed by DAVID gene ontology. The bars represent fold enrichment of each pathway and are shown in order of significance (*p* values) listed on the y axis. **(B)** Top 5 gene sets positively associated with SC144 treatment analyzed by GSEA. Gene sets with FDR *q*-value lower than 0.25 are considered true enrichment. **(C)** Enrichment plot of hallmark HYPOXIA gene set over-represented most significantly out of the pre-ranked gene lists from SC144 treatment in OVCAR-8 cells. Nascent RNA encoding hypoxia genes were the most upregulated in SC144-treatment compared to control in OVCAR-8 cells. Bar plot for fold change of relative transcription level of 142 genes in the HYPOXIA gene set from SC144 treatment compared to control in OVCAR-8 cells. **(D)** Enrichment plots of hallmark GLYCOLYSIS and MTORC1-SIGNALING gene set over-represented most significantly out of the pre-ranked gene lists from SC144 treatment in OVCAR-8 cells.

To identify the association between SC144 treatment and biological processes, gene set enrichment analysis was performed using DAVID KEGG gene sets and Molecular Signatures Database (MSigDB, GSEA - Broad Institute). SC144 upregulated genes functioning in a wide range of biological processes, including “cytokine signaling pathways”, “oncogenesis signaling”, “immune response”, “cell adhesion” and “migration” (Figure 1A). Furthermore, “cytokine-cytokine receptor interaction” and “chemokine signaling pathway” were positively enriched while “Systemic lupus erythematosus”, an autoimmune-induced inflammatory disease, and “Alzheimer's disease” were down-regulated gene sets. Systemic inflammation interferes with immunological processes of the brain and has been connected to the progression of this disease 63. These findings suggest that SC144 may play an important role in mediating inflammatory and immune signaling.

**Figure 2:**
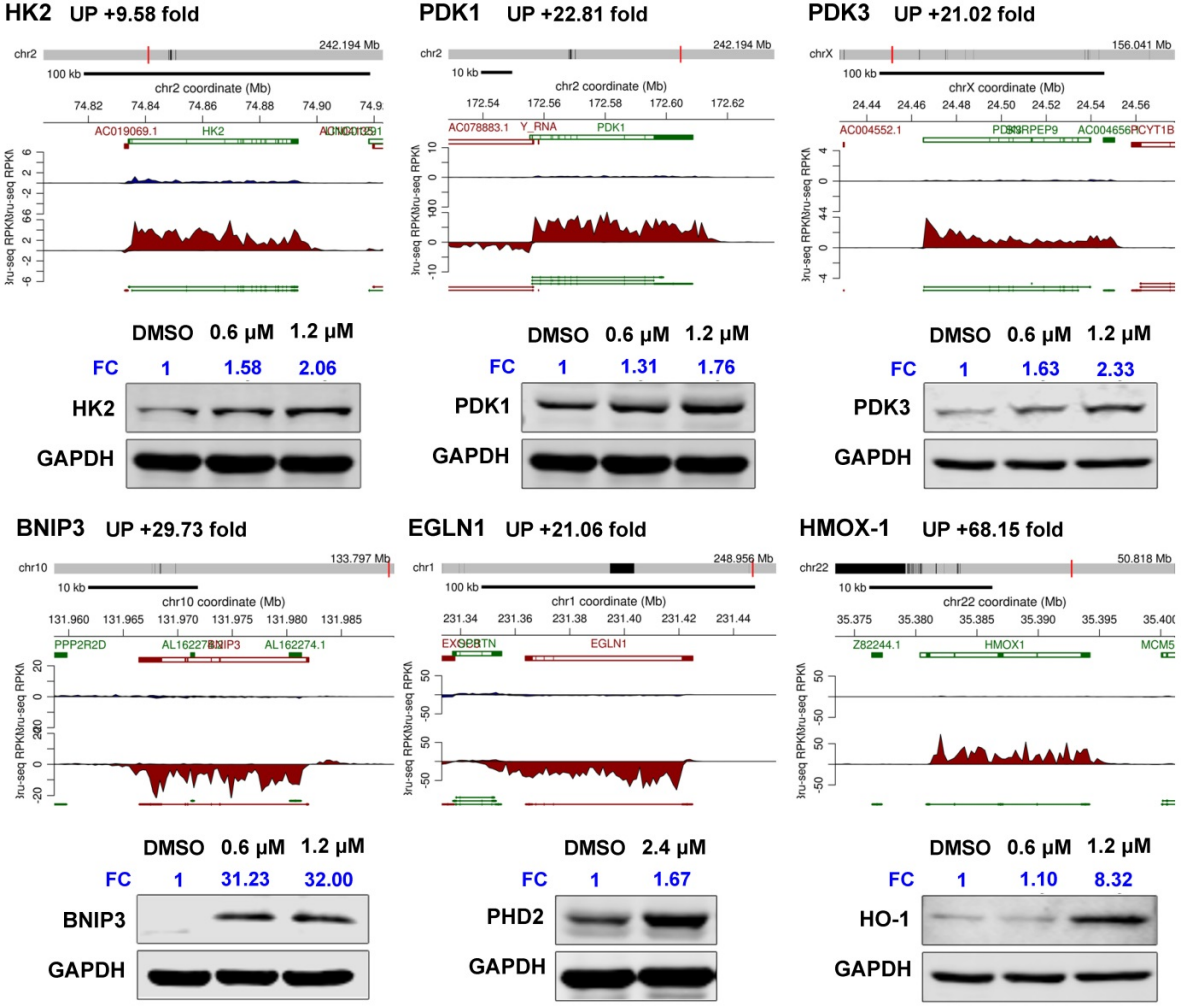
SC144 upregulates hypoxia-related mediators. Synthesis of HK2, PDK1/3, BNIP3, EGLN1 and HMOX-1 nascent RNAs are up-regulated by SC144 in OVCAR-8 cells as identified by Bru-Seq. OVCAR-8 cells were treated with 0.6 and 1.2 μM SC144 for 2 h, DMSO was used as vehicle control. Images are representative of 3 independent experiments. Statistical analysis is provided in Supplemental Figure S2. FC = fold change, the expression levels of proteins fold to DMSO-control cells.

GSEA revealed that gene sets related to “hypoxia”, “glycolysis”, “MTORC1 signaling” and immune response-related processes were significantly positively associated with SC144 treatment at the transcriptional level (FDR, *q*-value < 0.1, Figure 1B, Tables S13-14). We next assessed the transcription level of genes in the top 3 enriched gene sets. In response to SC144 treatment, 89/154 and 115/184 genes involved in glycolysis and activation of mTORC1 complex, respectively, were transcriptionally upregulated (Figures 1A, 1D and S1). Particularly, SC144 significantly up-regulated transcription of genes involved in hypoxia signaling (Figures 1B-C). In a set of 142 genes that are upregulated in response to hypoxia, 105 genes were transcriptionally increased upon exposure to SC144 (Figure 1C). We next determined the protein expression levels of several key hypoxia damage markers. Consistent with their elevated transcriptional levels, all tested proteins, HK2, PDK1/3, BNIP3, EGLN1, and HMOX-1, were upregulated upon SC144 treatment at different degree (Figure 2, Figure S2). BNIP3 was the most upregulated, with a fold change ~32 at 0.6-1.2μM SC144 treatment. We also used the Connectivity Map L1000 platform (Broad Institute) to examine potential similarities of SC144 with other small molecules or characterized genetic perturbations (Figures S3-4, Tables S19-20). We observed that SC144 treatment is positively correlated with caspase activators and hypoxia-inducible factor activators. Collectively, our data suggest that SC144 induces transcription of genes involved in hypoxic stress response in OC cells.

### SC144 transiently upregulates HIF1A antisense RNA

**Figure 3:**
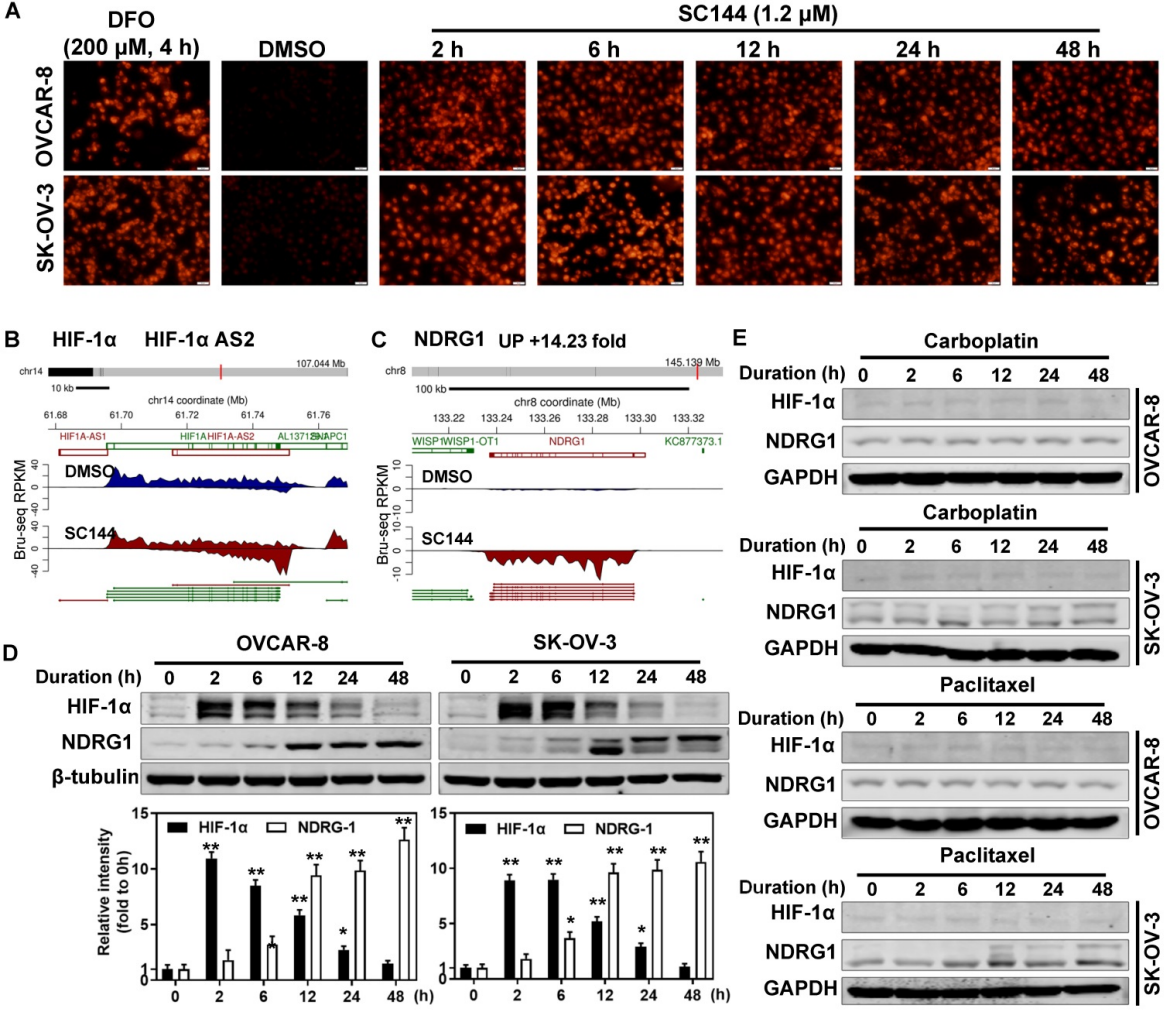
SC144 induces hypoxic stress and transiently upregulates HIF1 anti sense RNA. **(A)** OVCAR-8 and SK-OV-3 cells were treated with SC144 (1.2 μM) for 2, 6, 12, 24 and 48 h, then, the cell hypoxia levels were detected using a hypoxia kit. The red color represents nitroreductase activity in hypoxic cells. DMSO was used as vehicle control and DFO (200 μM) was used as a positive control. Images are representative of 3 independent experiments. Scale bar, 20 µm. **(B-C)** Synthesis of HIF1A-AS2 and NDRG1 nascent RNA are up-regulated by SC144 in OVCAR-8 cells. OVCAR-8 cells were treated with 2 μM SC144 for 4 h. The gene map is from RefSeq Genes (UCSC genome browser, http://genome.ucsc.edu/). **(D)** SC144 increases the protein expression levels of HIF-1α and NDRG1. OVCAR-8 and SK-OV-3 cells were treated with 1.2 μM SC144 for 2, 6, 12, 24 and 48 h. DMSO was used as vehicle control. Images are representative of 3 independent experiments. **(E)** Carboplatin and paclitaxel do not affect the protein expression levels of HIF-1α and NDRG1. OVCAR-8 and SK-OV-3 cells were treated with 10 μM carboplatin or 15 nM paclitaxel for 2, 6, 12, 24 and 48 h. DMSO was used as vehicle control. Images are representative of 3 independent experiments.

In hypoxic solid tumors, increased expression of nitroreductase is detected 64. Thus, we determined the hypoxia levels by measuring the nitroreductase activity of OC cells in response to SC144 treatments. 1.2 μM SC144 induced equivalent level of nitroreductase activities as 200 μM DFO in OVCAR-8 and SK-OV-3 cells starting as early as 2h (Figures 3A and S5A). Next, we assessed the expression of HIF-1α and key modulators of the transcriptional response to hypoxic stress. From Bru-seq analysis, though the transcription of HIF1A (HIF1α coding gene) is not affected by SC144, the transcription of HIF1A-AS2 was increased after 4 hours in OVCAR-8 cells (Figure 3B). A significant elevation of HIF-1α protein was observed in SC144 treated cells as early as 2h (9-fold in SK-OV-3 and 11-fold in OVCAR-8), and this elevation is dose dependent with 4-fold at 1.2 µM and 6-fold at 2.4 µM compared to control (Figure 3D and Figure S5B). A gradual decrease of HIF-1α protein level was observed at 12 to 48 h (Figure 3D). Similarly, SC144-induced changes of HIF-1α protein levels were also confirmed in a mouse ovarian ID8 and human LN-CaP cells (Figure S5C). Interestingly, Bru-seq analysis identified that N-MYC downstream regulated gene 1 (NDRG1, DRG-1), a stress reactive anti-cancer factor, was also transcriptionally up-regulated (14-fold) (Figure 3C). The protein level of NDRG1 was increased in a time-dependent manner in both cell lines (11-fold in SK-OV-3 and 13-fold in OVCAR-8, Figures 3D and S5C). Proteasome inhibitor, MG-132, pre-treatments (10 μM for 1-4h) had no effect on SC144-induced decrease of HIF-1α protein levels at 24 h, indicating that the diminished HIF-1α protein levels was not due to increased degradation through ubiquitin-proteasome (Figure S5D). To verify if induced hypoxic stress response are common response of cancer cells to chemotherapeutic treatments, we determined the expressions of HIF-1α and NDRG1 after exposure to carboplatin and paclitaxel. As expected, the expression of HIF1A and NDRG1 were not affected by carboplatin and paclitaxel in OVCAR-8 and SK-OV-3 cells (Figure 3E). Cumulatively, our data suggest that SC144 induces transient up-regulation of HIF-1α protein expression and up-regulating the transcription of HIF1A-AS2 and NDRG1.

### SC144 up-regulates protein expression of NDRG-1 and its downstream signaling

**Figure 4:**
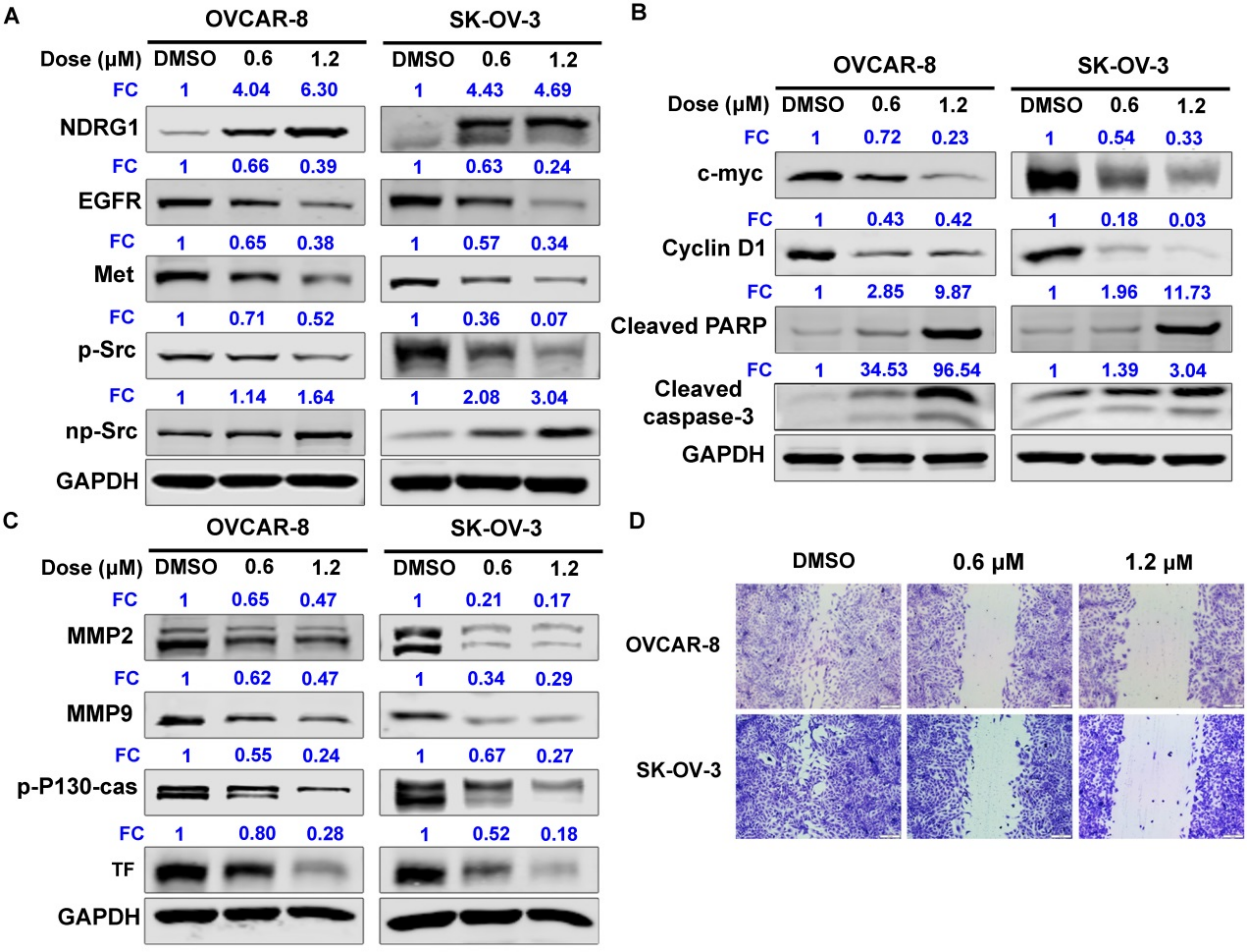
SC144 up-regulates the protein expression levels of NDRG-1 and its select downstream signalling genes. (**A)** SC144 up-regulates NDRG-1 protein expression levels and EGFR/Met mediate Src signaling. OVCAR-8 and SK-OV-3 cells were treated with SC144 (0.6 and 1.2 μM) for 48 h, and blotted with antibodies to NDRG1, EGFR, Met, Src and p-Src. (**B)** SC144 increases the protein expression levels of pro-proliferative factors and decreases the cleavages of pro-apoptotic proteins. OVCAR-8 and SK-OV-3 cells were treated with SC144 (0.6 and 1.2 μM) for 48 h, then, c-MYC and cyclin D1 protein levels were determined as pro-proliferative factors and cleaved-PARP and cleaved-caspase-3 were measured as pro-apoptotic proteins. (**C)** SC144 suppresses invasion, migration and angiogenesis related protein expressions. OVCAR-8 and SK-OV-3 cells were treated with SC144 (0.6 and 1.2 μM) for 48 h and blotted with antibodies to MMP2 and MMP9 (pro-invasion markers), p-P130-cas (migration marker) and TF (pro-angiogenesis marker). DMSO was used as vehicle control. **(D)** SC144 inhibits cell migration. OVCAR-8 and SK-OV-3 cells were treated with SC144 (0.6 and 1.2 μM) in RPMI 1640 with 1% FBS for 24 h. Then, cells were stained with crystal violet solution (2%) and scratches were imaged using a microscope. Images are representative of 3 independent experiments. FC = fold change, the expression levels of proteins fold to DMSO-control cells.

NDRG1 acts as a potent tumor suppressor by inhibiting signaling pathways involved in mediating cell proliferation, migration, invasion, and angiogenesis. We further observed that SC144 increased NDRG1 protein expression of OC cells in a dose-dependent manner: 4-fold at 0.6 µM and 6-fold at 1.2 µM compared to control in OVCAR-8 cells (Figure 4A and S6A). In contrast, SC144 decreased the protein levels of EGFR and Met in a dose- and time-dependent manner with ~0.3-fold at 1.2 µM after 48h treatment (Figures 4A and S6). Treatment with SC144 for 48 hours significantly reduced phosphorylation of Src in both OVCAR-8 and SK-OV-3 cells (Figures 4A and S6A). As shown in Figure 4B and S6A, SC144 suppressed protein expressions of pro-proliferative proteins, c-MYC (by ~0.3-fold at 1.2 µM), and cyclin D1 (by 0.03-0.4-fold at 1.2 µM), in a dose-dependent manner at 48h in both cell lines. Meanwhile, a significant increase of pro-apoptotic proteins, cleavaged PARP and caspase-3, were observed in OC cells when treated by 1.2 µM SC144 for 48h, indicating that cells were undergoing apoptosis after SC144 treatment. MMP2 and MMP-9 are matrix metalloproteinases that promote the invasion of cancer cells. p-P130-cas plays an important role in cancer cell migration, and Tissue factor (TF) is a vital pro-angiogenesis factor in cancer. SC144 significantly decreased the protein levels of MMP2, MMP-9, p-P130-cas and TF in OVCAR-8 and SK-OV-3 cells (Figures 4C and S6), leading to decreased cell migrations (Figure 4D).

### SC144 sensitizes ovarian cancer cells to chemotherapeutic agents

**Figure 5:**
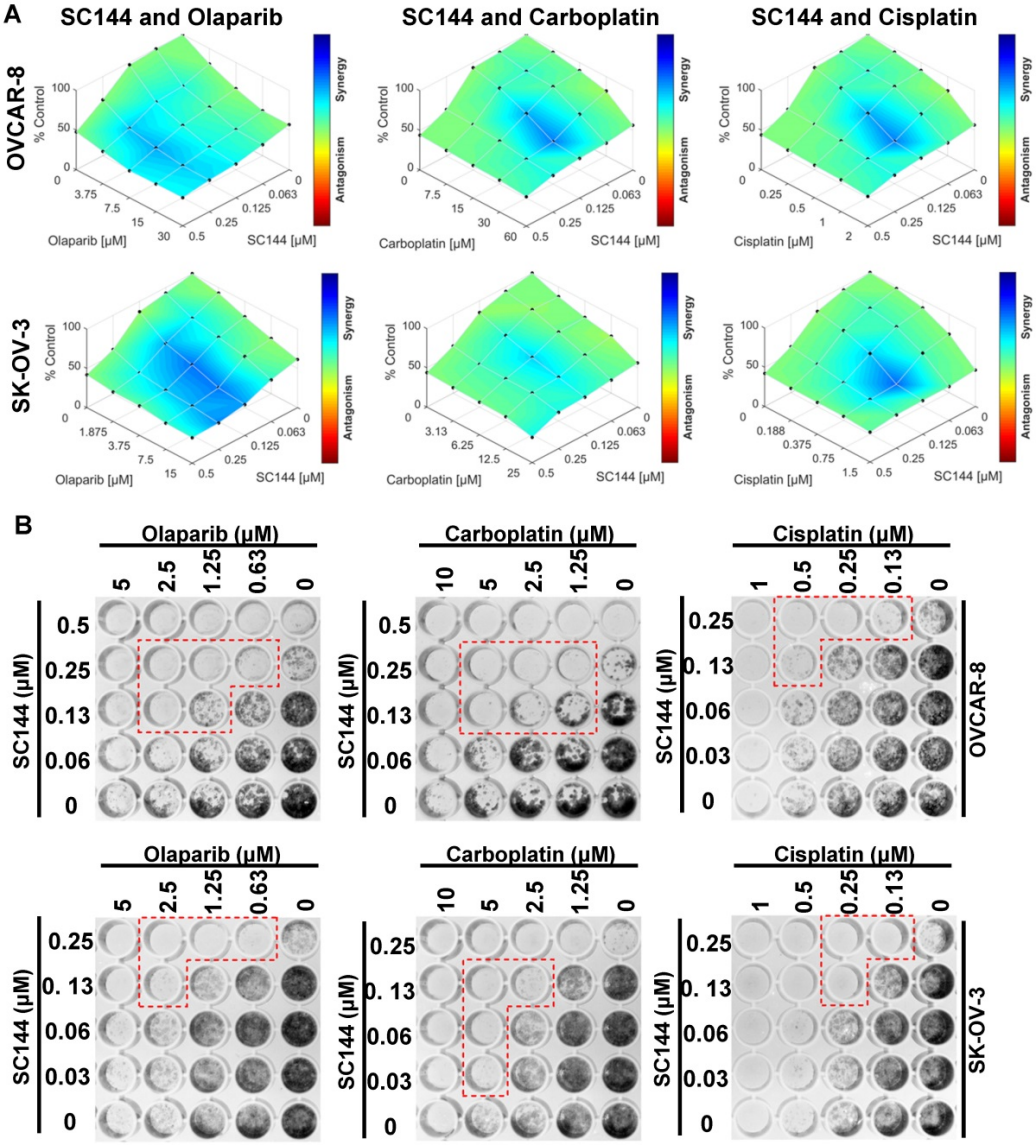
SC144 shows synergistic effects with chemotherapeutic agents in 2D culture. (**A)** OVCAR-8 and SK-OV-3 cells were treated with the combinations of SC144 and olaparib, carboplatin and cisplatin for 72 h. Cell viabilities were determined using MTT assay and Combenefit software (v2.02) was used for combination analysis with the HSA model. (**B)** The synergistic effect of SC144 with chemotherapeutic agents was observed in colony formation assays. OVCAR-8 and SK-OV-3 cells were treated with the combinations of SC144 and olaparib, carboplatin and cisplatin for 7 d, then, colonies were stained and imaged. Red lines indicate wells with significant synergistic effect from the combination treatment. Images are representative of 3 independent experiments.

**Figure 6:**
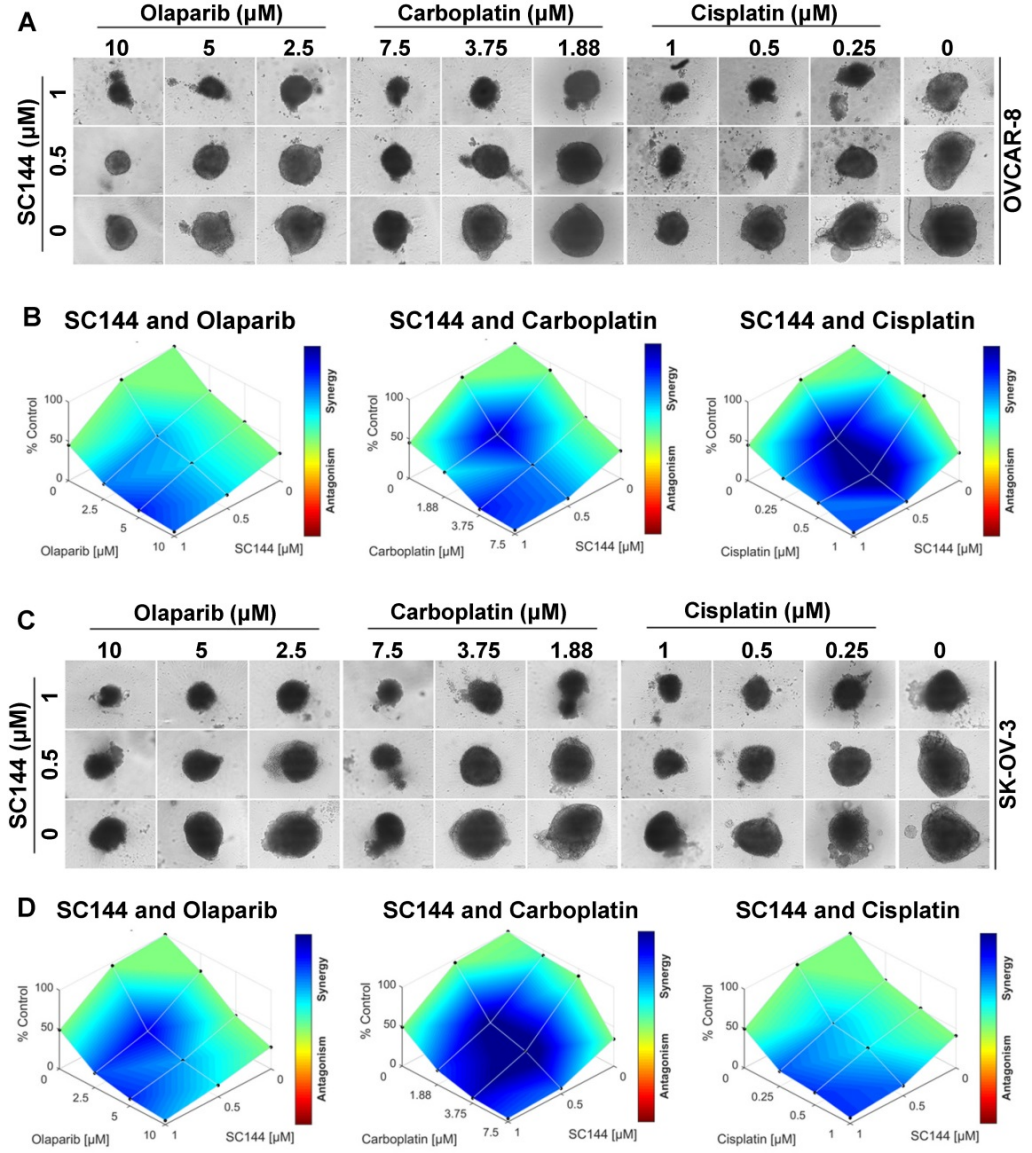
SC144 shows synergistic effects with chemotherapeutic agents in 3D culture. (**A,C)** SC144 increases the effect of chemotherapeutic agents in 3D culture of OVCAR-8 and SK-OV-3 cells. The spheroids of OVCAR-8 and SK-OV-3 cells were treated with the combinations of SC144 and olaparib, carboplatin and cisplatin for 7 d, then, the spheroids were imaged. (**B, D)** Combination analysis of SC144 and chemotherapeutic agents. Cell viability of the spheroids was measured with the CellTiter-Glo® 3D Cell Viability Assay (Promega). The luminescence reading of spheroids in each treatment were compared to the control spheroids and calculated using Combenefit software (v2.02) with HSA model. Scale bar, 100 μm. Images are representative of 3 independent experiments.

Next, we investigated the potential for synergy with SC144 by combining SC144 with platinum-based drugs, carboplatin, cisplatin, and PARP inhibitor, olaparib. As observed in MTT and colony formation assays, SC144 alone and in combination with carboplatin, cisplatin and olaparib greatly inhibited growth in OVCAR-8 and SK-OV-3 cells. Synergistic effects were observed at various concentrations (Figure 5A-B). Similar synergistic effects were observed in ID8 cells (Figure S7). We next tested the combination treatments of SC144 in a 3D spheroid model with OVCAR-8 and SK-OV-3 cells. The sizes of spheroids in wells containing both drugs including the combination of SC144 and olaparib, SC144 and carboplatin, SC144 and cisplatin, were smaller than those treated with each individual agent (Figure 6A and C). Notably, the synergistic effects were more pronounced in 3D spheroid culture than the 2D cultures (Figure 6B and D). Collectively, these data demonstrate that SC144 sensitizes OC cells to olaparib, carboplatin and cisplatin in both 2D and 3D cultures, suggesting the utility of combination of SC144 and chemotherapeutic agents as an effective therapy for OC.

### SC144 induces comparable cellular transcriptional profiling as CPX and exhibits metal-mediated cell kill

**Figure 7:**
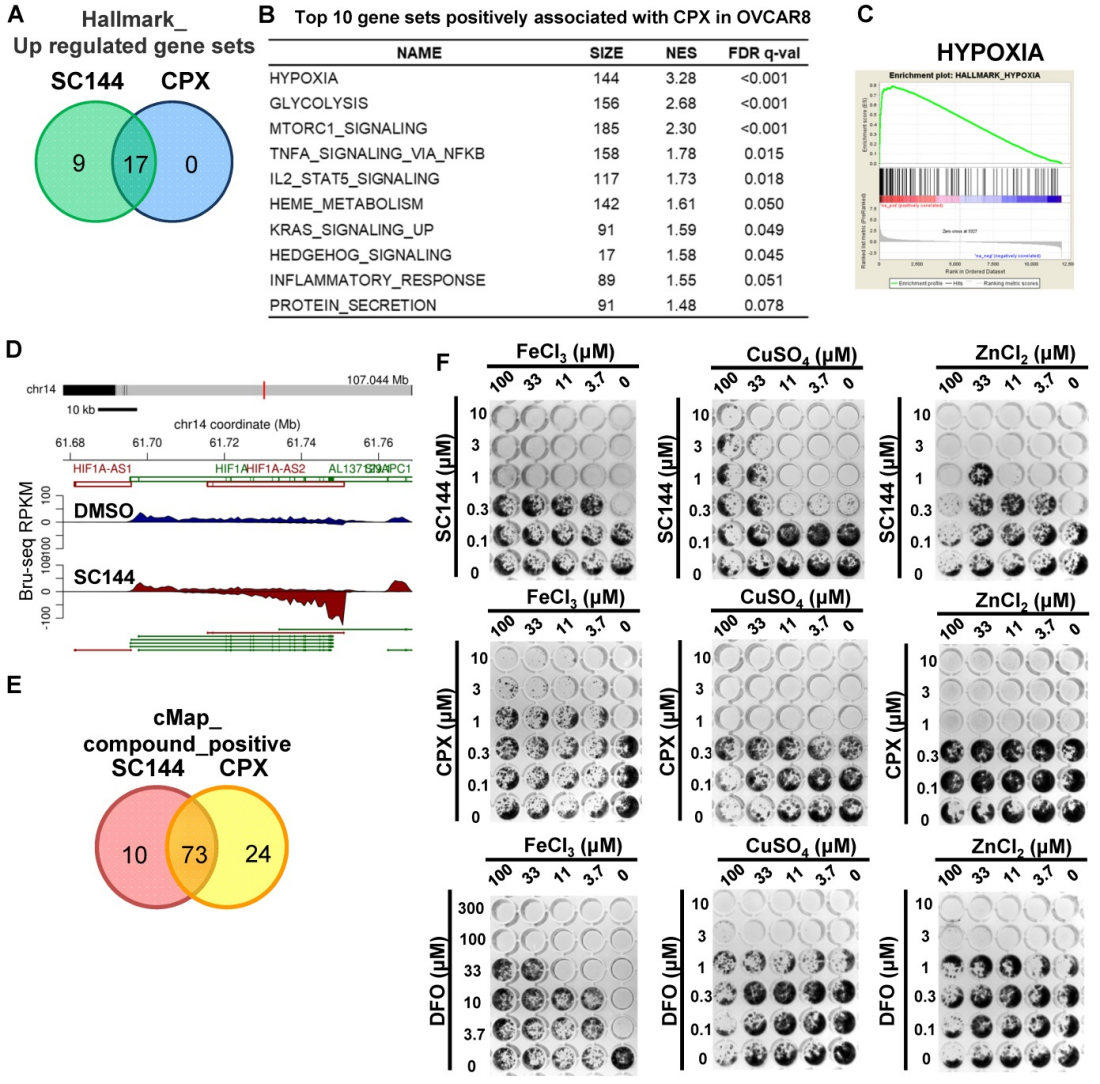
CPX induces comparable cellular transcriptional profiling as SC144. **(A)** Top upregulated gene set in common between SC144 and CPX treatment in OVCAR-8 cells. Gene sets with FDR *q*-value lower than 0.25 were considered as true enrichment. **(B)** Top 10 gene sets positively associated with CPX treatment in OVCAR-8 cells. **(C)** Enrichment plots of hallmark HYPOXIA gene set over-represented most significantly out of the pre-ranked gene lists from CPX treatment in OVCAR-8 cells. **(D)** Synthesis of HIF1A-AS2 nascent RNA is up-regulated by CPX in OVCAR-8 cells as identified by Bru-Seq. **(E)** Overlapping of positively correlated compounds with SC144 and CPX analysed by CMap in OVCAR-8 cells. Compounds with a median score >90 are considered. **(F)** The antiproliferative effects of SC144, CPX and DFO were rescued by Fe, Cu and Zn in a dose-dependent manner. OVCAR-8 cells were treated with SC144, CPX and DFO. FeCl_3_, CuSO_4_ and ZnCl_2_ were added together with the compounds at indicated concentrations. Cell viabilities were determined using colony formation assays.

To gain additional mechanistic insights, we compared Bru-seq results of 200 diverse compounds developed in our laboratory and various clinically used drugs (data not shown). An antifungal compound, CPX, was found to show a profile that clustered with the profile induced by SC144 (Tables S5-8). Gene set enrichment analysis revealed that 17/17 gene sets were significantly upregulated by CPX and SC144 treatment (Figure 7A, Table S15-16). Top gene sets up-regulated by CPX at the transcriptional level included “hypoxia”, “glycolysis”, “MTORC1 signaling” and “immune response-related processes” (Figure 7B). Notably, CPX significantly up-regulated transcription of genes involved in hypoxia signaling (Figure 7C, FDR q-value<0.001) as well as increased transcription of HIF1A-AS2 (Figure 7D). Meanwhile, CMap analysis revealed that a large overlap between CPX and SC144 showed a positive correlation, using a median score greater than 90 (Figure 7E, Table S21-22). These observations indicate that CPX induces similar transcriptional profiling as SC144, suggesting a similar cellular mechanism of action.

CPX was reported as a chelating agent and induces HIF-1α in normoxic cells. Cancer cell proliferation can be inhibited by iron chelating agents. Since SC144 was observed with similar transcriptional profiling as CPX, we evaluated and compared the compounds' cytotoxicity using metal (iron, copper and zinc) rescue in OVCAR-8 and SK-OV-3 cells. FeCl_3_, CuSO_4_ and ZnCl_2_ were added together with the compounds at indicated concentrations (Figures 7F and S8). We observed that ferric ion rescued the antiproliferative effects of SC144, CPX and DFO in a dose-dependent manner. Interestingly, cell growth inhibited by SC144 could be rescued by addition of CuSO_4_ and ZnCl_2_, while these metals had no effect on the cytotoxicity of DFO and CPX. These observations suggest that unique metal-dependent properties of these agents are partly responsible for their different cytotoxicity and mechanism of action.

### SC144 induces a hypoxic stress transcriptional response in LNCaP cells

**Figure 8:**
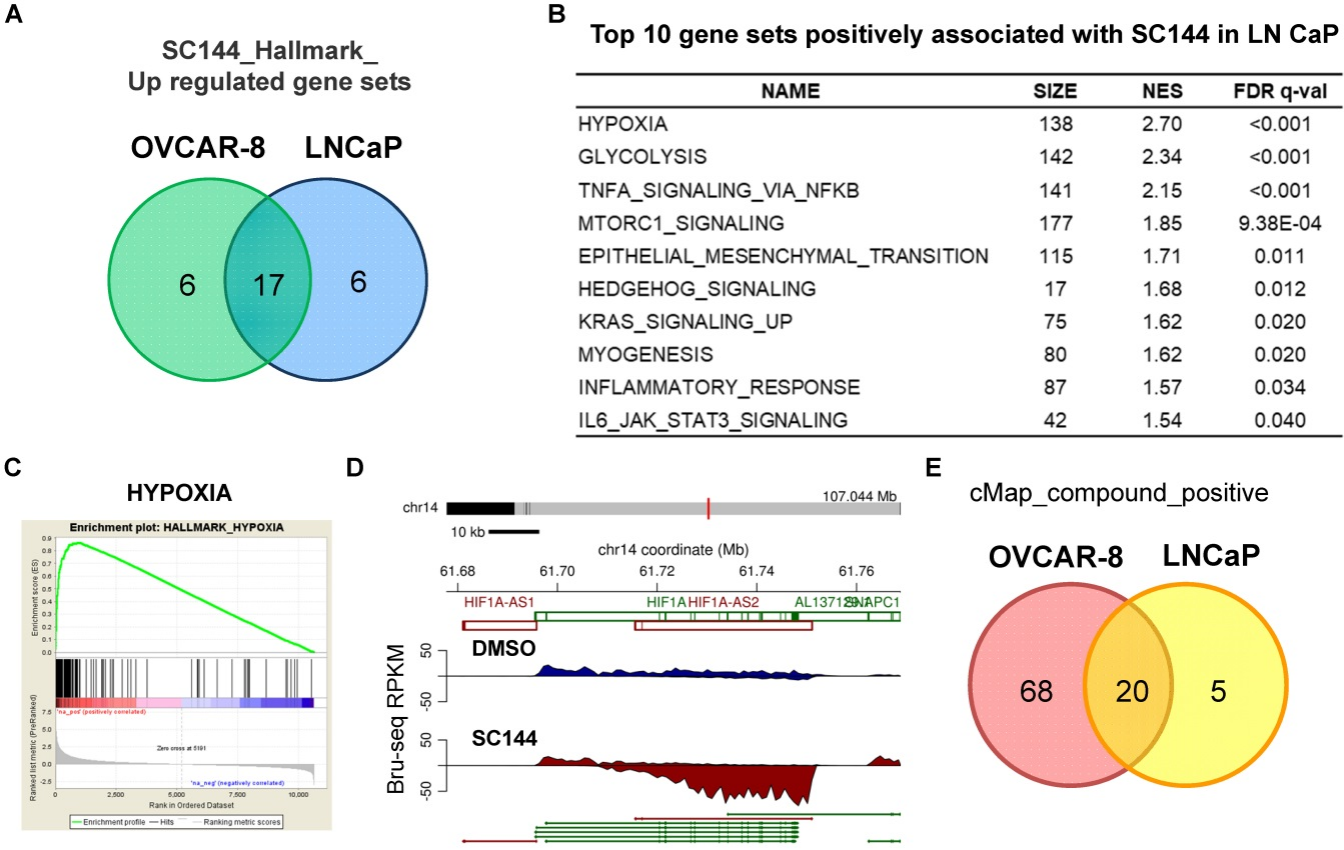
SC144 induces hypoxia in LNCaP cells. **(A)** Top 25 upregulated gene set overlapping by SC144 between OVCAR-8 and LNCaP cells. **(B)** Top 10 gene sets positively associated with SC144 treatment in LNCaP cells. Gene sets with FDR *q*-value lower than 0.25 are considered true enrichment. **(C)** Enrichment plots of hallmark HYPOXIA gene set over-represented most significantly out of the pre-ranked gene lists from SC144 treatment in LNCaP cells. **(D)** Synthesis of HIF1A-AS2 nascent RNA is up-regulated by SC144 in LNCaP cells. **(E)** Overlapping of positively correlated compounds with SC144 analysed by cMap between SC144 OVCAR-8 and LNCaP cells. Compounds with a median score >90 are considered.

To rule out cell line-specific transcriptional effects by SC144, we performed similar experiments using the prostate cancer cell line LNCaP (Tables S9-12). Using ± 2-fold change as cut-off, we identified 218 up-regulated and 33 down-regulated genes in common between SC144 treated OVCAR-8 and LNCaP cells (Figure S9A). Transcriptional levels of nine (PPP1R3C, BNIP3, RIMKLA, PDK3, MT1X, C4orf47, RAB20, ANGPTL4 and DTNA) out of the top 25 genes were increased in both cell lines (Figure S10, Tables S1 and S9). Using the FDR, q-value of 0.25 as the cut-off, we observed that 17 genes were positively regulated by SC144 in both cell lines (Figures 8A and S9B, Tables S13 and S17). Consistent with the observation in OVCAR-8 cells, gene sets such as “hypoxia”, “glycolysis” and “MTORC1 signalling” were also positively enriched in LNCaP cells by SC144 at the transcriptional level (Figure 8B-C). We also observed that the transcription of HIF1A-AS2 was increased to 17-fold by SC144 compared to control after 4 hours treatment (Figure 8D). In addition, using a median score greater than 90, CMap analysis revealed 20 small molecules to be positively correlated to SC144 in both cell lines (Figures 8E and S11, Tables S23-24). Collectively, our data suggest that SC144 transcriptionally induces genes involved in hypoxia signalling in an independent cell line.

### SC144 delays tumor growth in two syngeneic models

**Figure 9:**
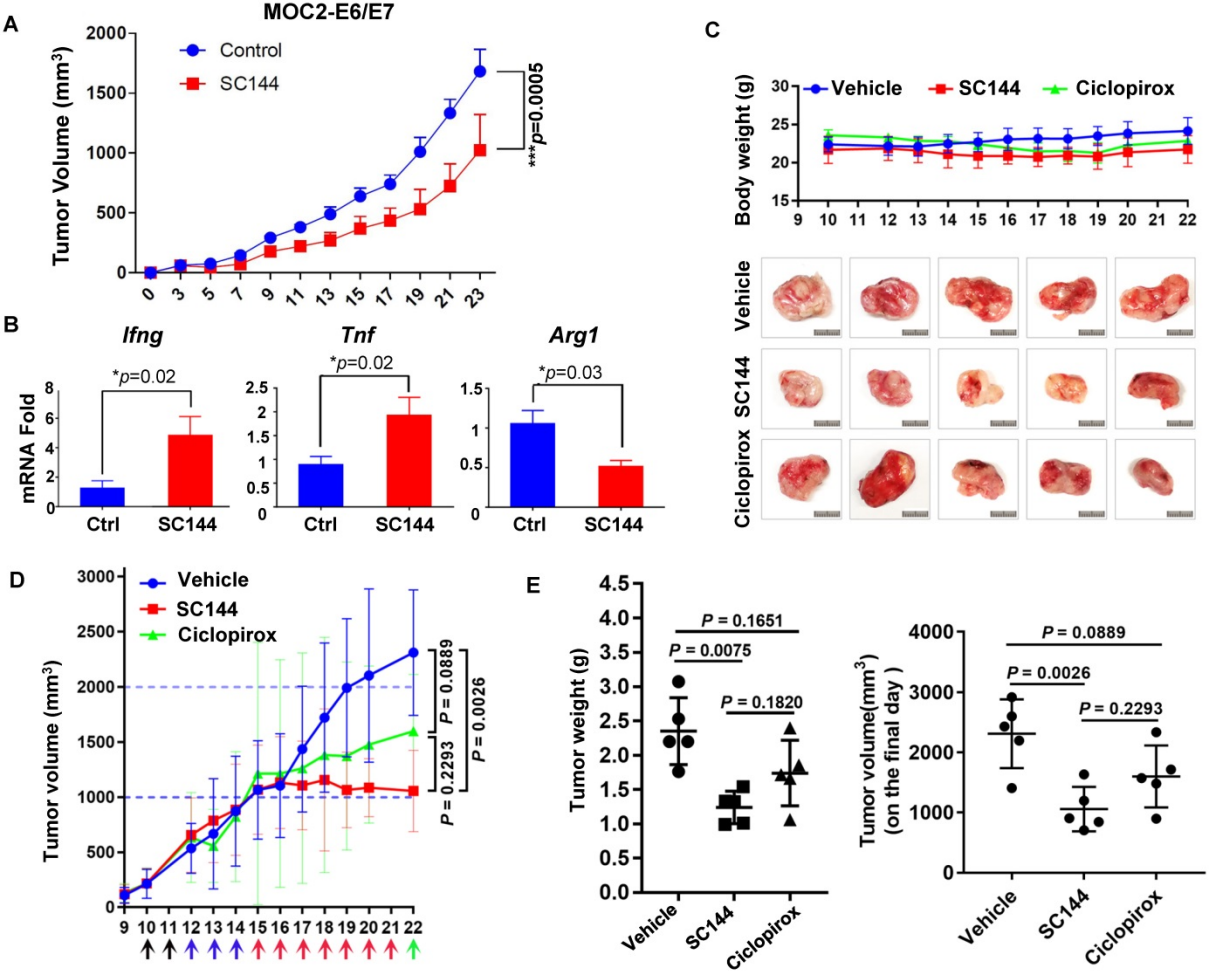
SC144 delays tumor growth in syngeneic mouse models. (**A)** Growth curves of s.c. MOC2-E6/E7 tumor-bearing C57BL/6 mice daily treated with vehicle or SC144. PBS or SC144 were administered on day 3 post-tumor implantation. Results represent mean ± SEM (n=7 for each group). Comparisons were made using a generalized estimating equation model. (**B)** Tumors from each group were harvested and homogenized for RNA extraction. Quantitative PCR (qPCR) was performed for Ifng, Tnf, and Arg1 (n=5 for each group). Results represent mean ± SEM, qPCR was performed in triplicates. Growth curves of body weight of mice treated with vehicle, SC144 or CPX. The body weight of mice was recorded daily, there were no significant changes in body weights of mice in each group (n=5). (**C)** Images of bulk tumors from CT-26 tumor-bearing BALB/c mice in control, SC144 or CPX-treated group. Scale bar, 1 cm. (**D)** Growth curves of s.c. CT-26 tumor-bearing mice daily treated with vehicle, SC144 or CPX. (**E)** The tumor weight and the tumors volume on the final day in SC144 group was significantly lower than vehicle-control, while CPX group was not. Mice were euthanized and tumors were isolated and weighed. Statistical significance was calculated using Student's t-test. Error bars indicate mean ± SEM (standard error of mean). *p < 0.05, **p < 0.01.

In a previous study we observed significant *in vivo* efficacy of SC144 in an immune compromised mice model of OC 42. In this study, we tested the efficacy of SC144 in immunocompetent mice models. We previously observed significant immune-regulatory genes upon SC144 treatment. Unfortunately, we were unable to produce consistent tumors in an ID8 syngeneic model of OC. Since there are not many effective and robust syngeneic mouse models of OC, we tested SC144 in two different syngeneic models of oral and colon cancers. The syngeneic mouse oral cancer (MOC2)-derived tumor models are highly aggressive and resistant to chemotherapy and checkpoint blockade immunotherapy, with suppressed inflammatory signatures 45, 65-67. SC144 as a single-agent showed significant reduction in tumor burden (Figure 9A). Notably, SC144 treated tumors displayed greater expression of immune effector signatures Ifng and Tnf, in addition to lower levels of arginase 1 (Arg1), a key marker of M2-like macrophage differentiation (Figure 9B). To further evaluate the *in vivo* efficacy of SC144 in other cancer models, we tested its effect on established tumors after subcutaneous inoculation of mouse colon cancer CT-26 cells in the flank of BALB/c mice. CPX was used as a positive control in this experiment. Compared with mice in the control group, no substantial body weight loss was detected in the treatment groups during the study (Figure 9C), indicating that SC144 did not exert significant adverse effects in mice at its effective dose. Compared with vehicle control treatment, i.p. administration of SC144 for 14 days significantly delayed tumor growth (Figure 9D-E). The tumor weights of mice in the SC144 group were significantly lower than those in vehicle group (Figure 9F). There were no significant differences in tumor volume and tumor weight of mice when comparing the CPX group and the vehicle group. Collectively, these results suggest that SC144 is a promising agent for cold tumors (MOC2-E6/E7) as well as hot tumors (CT-26).

## Discussion

Previously, we reported that SC144 binds gp130, induces gp130 phosphorylation and inhibits the expression of downstream target genes of the gp130 pathway in OC cells. In this study, we used genome-wide nascent RNA Bru-seq analysis to fully capture the nascent transcriptional signature induced by SC144 in cells within 4 hours of treatment. This study provides the first evidence that SC144 remarkably induces transcription of genes involved with hypoxic signaling and increases the expression of the non-coding RNA HIF1A-AS2.

A series of cellular adaptations promote cancer cells survival under hypoxic conditions. The hypoxic adaptation is mainly ascribed to hypoxia-inducible genes and proteins. A wide range of hypoxia-related genes and proteins are reported to be overexpressed in tumor samples from OC patients 68, 69. Moreover, in OC patients with strong expression of HIF-1α in their tumors, have a significantly shorter overall survival 69. Therefore, targeting hypoxia, especially HIF-1α, and its upstream signaling partners or downstream effectors is a promising approach to treat OC and several other cancers. HIF inhibitors block the expression and/or functions of HIF-1α and/or HIF-2α through direct or indirect mechanisms, including the inhibition of the mRNA expression (e.g. PX-748) 13, protein synthesis (e.g. 2ME2) 15, protein accumulation and stabilization (e.g. bisphenol A) 70, dimerization (e.g. cyclo-CLLFVY) 14 and DNA binding (e.g. echinomycin) 71. In addition to HIFs, the antisense RNAs of HIFs are also promising targets for cancer therapy. HIF-AS inhibit the translation of HIFs and improve the anticancer action of cytotoxic drugs 72. Previously, it was shown that HIF1A-AS downregulates the expression of HIF-1α and enhances tumor immunotherapy in a syngeneic mouse model 73. HIF1A-AS also suppresses tumor growth, angiogenesis and cell proliferation, and induces apoptosis in a liver cancer model 74. EZN-2968, a synthetic antisense oligodeoxynucleotide, was evaluated in phase I clinical trials (NCT01120288, NCT00466583) and showed preliminary benefit in cancer patients, but the study was terminated 18, 75. In this study, we explored the effect and underlying mechanism of SC144 induction of hypoxic responses and cell death in OC cells. Interestingly, the protein expressions of NDRG1 and its downstream anti-cancer signaling are upregulated in OC. The expression of HIF-1α transiently increased, and gradually decreased over time, possibly due to the elevated transcription of HIF1A-AS2. Notably, SC144 treatment resulted in 500-fold upregulation of HOXA13 as well as a 68-fold upregulation of HMOX1. HOXA13 is linked to poor prognosis in many cancers and HMOX1 has a role in protecting tumor cells from apoptosis. A possible explanation is that cells under SC144 pressure need to upregulate these genes to survive. After 4 hours of drug treatment, the expression of numerous protective genes went up as cells try to counteract death. However, eventually cells die and the tumor size decreases.

Select iron chelators show antiproliferative properties due to direct and indirect actions on a wide range of crucial metabolic pathways, including DNA synthesis and ATP production 76. DFO and CoCl_2_ can induce hypoxic stress and up-regulate HIF-1α in a variety of cell types in a dose and time-dependent manner 68, 77. In this study, we observed that SC144 is significantly more cytotoxic than DFO and CoCl_2_. A previous study performed whole-genome gene array on MCF-7 cells treated with DFO and compound 311, a reported iron chelator 76, and identified genes up- and down-regulated by iron chelation 78. Interestingly, 22 out of the 30 upregulated genes and 10 out of the 21 downregulated genes from this study have also been observed by us to be transcriptionally affected in the same direction by SC144. Notably, SC144 significantly increased NDRG1 at the transcriptional and protein level. NDRG1 expression is very low in many tumor types and inhibits metastatic progression in patients with prostate cancer 79. Similar to our observations, NDRG1 was specifically upregulated by iron depletion 80. Knocking out of HIF-1α in murine embryo fibroblasts reduced NDRG1 upregulation by DFO, revealing a HIF-1α-dependent mechanism 80. Collectively, our data show that SC144 shares a potential gene signature of iron chelation similar to CPX.

In the CNS, iron accumulation in the brain induces oxidative stress during aging and is a hallmark of many neurodegenerative processes 81, 82. Multiple iron chelators are under development as neuroprotective, for treating neurodegenerative disorders, such as Alzheimer's, Parkinson's, multiple sclerosis and Huntington's diseases 83, 84. In our study, functional enrichment analysis revealed that “Alzheimer's diseases” “Huntington's diseases” and “Parkinson's diseases” are among the top 10 negatively enriched gene sets by SC144. These observations suggest a tight correlation between SC144 and iron homeostasis and a possible neuroprotective effect of SC144 in select CNS diseases.

Safety and efficacy of iron chelators have been assessed in various clinical trials. DFO exhibited selective cytotoxic effect on neuroblastoma cells over normal bone marrow cells in phase II clinical studies. Seven of nine patients responded with up to 50 % reduction in bone marrow infiltration after a single course of DFO administered i.v. for 5 days at 150 mg/kg/day 85. In a study of 10 patients with hepatocellular carcinoma, the overall response rate to DFO was 20% and one patient with a massive tumor and lung metastases responded well 38. However, due to its highly hydrophilic nature, DFO has to be administered parenterally and has clinical use limitations, such as poor gastrointestinal absorption, short plasma half-life (12 min) and hematological toxicity 86, 87. It has been reported that DFO treatment was insufficient to reduce the cardiac iron burden and had low compliance in patients 88. Antibodies, such as ^89^Zr-DFO-Trastuzumab and ^89^Zr-DFO-MSTP2109A, are currently undergoing phase I/II clinical trials for esophagogastric and prostate cancer treatment 89. Deferiprone (DFP) and DFX, second generation DFO analogs, are lipophilic and orally bioavailable. Although both agents have good compliance, DFP has a poor safety profile, while DFX has a reasonable safety as compared to DFO. Serious side effects of DFX include gastrointestinal disturbances, arthropathy, neutropenia and agranulocytosis 87.

Connectivity Map (cMAP) analysis characterizes the mechanism of action of a small molecule-based on similar profiles of compounds with known function 50. Our cMAP analysis of Bru-seq data show that compounds that share similar cellular signatures with SC144 are involved in many biological responses, including apoptosis induction (e.g. TW-37, PAC-1), hypoxia activation (e.g. VU-0418947-2, cobalt(II)-chloride), matrix metalloproteinase inhibition (e.g. WAY-170523, UK-356618), STAT3 suppression (e.g. pyrrolidine dithiocarbamate, cucurbitacin-I) and iron chelation (hinokitiol). Hinokitiol was identified as the second top compound that positively correlated to SC144. Also known as β-thujaplicin, hinokitiol is a natural tropolone derivative. Due to the α-diketone fragment, it can chelate iron and induce apoptosis 90. Moreover, hinokitiol increased transcriptionally active HIF-1α via inhibition of HIF-specific hydroxylases 91. In well-oxygenated environments, HIFs are inactivated by prolyl hydroxylases (PHDs) and asparagine hydroxylase (factor-inhibiting HIF, FIH), that target HIF for ubiquitin-proteasome degradation. Hypoxic stress diminished PHDs activities, leading to the stabilization of HIFs. In turn, the mRNA levels of PHDs, especially PHD1 and 3, are upregulated by HIF1α through a PHD-HIF feedback loop in various cell types 92-94. Interestingly, though we observed that hypoxia signaling is dramatically upregulated after exposure to SC144, the transcription of EGLN1 and 3 (PHD2 and PHD3 coding gene, respectively) are among the top upregulated genes in OVCAR-8 (21.06 and 5.91-fold change, respectively) and LN-CaP (9.05 and 78.91-fold change, respectively) cells. These increased gene levels could be due to feedback regulation of cells under hypoxia.

This study describes for the first time that SC144 induces a cellular transcriptional profile comparable to CPX. The antimicrobial CPX is approved to treat cutaneous fungal infections. It shows anticancer activity through binding to intracellular iron, disrupting cell division signaling and inducing apoptosis 95, 96. Therefore, CPX has been repositioned as an anti-cancer agent, and a phase I trial of oral CPX in patients with hematologic malignancies has been completed 41, 97. Moderate hematologic improvement was observed, while dose-limiting toxicity was observed at 80 mg/m^2^ 97. In addition, prior toxicology data indicated significant degenerative changes in heart, liver and lungs in rats, guinea pigs, rabbits and dogs 41. Due to the poor oral bioavailability, gastrointestinal toxicity, and poor water solubility, a prodrug of CPX, CPX-POM, is currently under phase I clinical trial for the treatment of advanced bladder tumors 98. Our Bru-seq analysis reveals a similar transcriptional signature between CPX with SC144. Our previous study has shown that SC144 significantly delays tumor growth in human OC xenografts with no substantial toxicity observed at effective anticancer dosage 42. In this study, we did not observe toxicity of SC144 even at i.p doses as high as 100 mg/kg given for over 30 days. On the basis of such observations, SC144 could potentially be useful for clinical studies. Importantly SC144 sensitizes OC cells to chemotherapeutic agents by exhibiting synergistic effect with platinum compounds and olaparib.

In conclusion, SC144 represents a novel anti-cancer drug that induces transcription of genes involved in hypoxic stress, up-regulation of HIF1A-AS2, and activates a series of important signaling events responsible for cytotoxicity and immune activation. This study provides significant new insights and further supports the potential clinical use of SC144 in OC patients.

## Supplementary Material

Supplementary figures and tables.

## Abbreviations

Bru-Seq: bromouridine sequencing
DAVID: database for annotation, visualization and integrated discovery
FDR: false discovery rate
GSEA: gene set enrichment analysis
HIFs: hypoxia-inducible factors
lncRNA: long non-coding RNA
ncRNA: non-coding RNA
OC: ovarian cancer
RPKM: reads per kilobase of transcript per million mapped reads
SD: standard deviation
