# Supplementary material for: Up-regulation of hypoxia-inducible factor antisense as a novel approach to treat ovarian cancer
Source: Theranostics. 2020 May 25;10(15):6959–76. doi: 10.7150/thno.41792 (PMC7295058; doi:10.7150/thno.41792)
Supplement: Supplementary file 1 — Supplementary figures and tables. [file thnov10p6959s1.pdf]

Up-regulation of hypoxia-inducible factor antisense as a novel approach to treat ovarian cancer

Tiangong Lu[1,ϕ], Jianming Tang[1,2,ϕ], Binita Shrestha[1], Blake R. Heath[3], Li Hong[2], Yu L. Lei[3], Mats Ljungman[4], and Nouri Neamati[1✉]

1. Department of Medicinal Chemistry, College of Pharmacy, Rogel Cancer Center, University of Michigan, Ann Arbor, MI 48109-2800, USA
2. Department of Gynecology and Obstetrics, Renmin Hospital of Wuhan University, 238 Jiefang Road, Wuhan, Hubei province, 430060, P.R. China
3. Departments of Periodontics and Oral Medicine and Otolaryngology- Head and Neck Surgery, Rogel Cancer Center, University of Michigan, Ann Arbor, MI 48109-2800, USA
4. Department of Radiation Oncology, University of Michigan Medical School, Rogel Cancer Center and Department of Environmental Health Sciences, School of Public Health, University of Michigan, Ann Arbor, MI 48109-2800, USA

✉ Corresponding author: N.N. E-mail: neamati@umich.edu; Phone: 734-647-2732

ϕ Contributed equally to this work

Supplementary figure titles and legends

Figure S1: SC144 induces hypoxia signaling. (A) Bar plot for fold change of relative transcription level of 154 and 184 genes in hallmark GLYCOLYSIS and MTORC1-SIGNALING gene sets from SC144 treatment compared to control in OVCAR-8 cells. (B) Enrichment plots of hallmark TNFA_SIGNALING_VIA_NFKB, INFLAMMATORY_RESPONSE, IL6_JAK_STAT3_SIGNALING, IL2_STAT5_SIGNALING, COMPLEMENT, REACTIVE_OXIGEN_SPECIES_PATHWAY and KRAS_SIGNALING_UP gene sets over-represented most significantly out of the pre-ranked gene lists from SC144 treatment in OVCAR-8 cells.

Figure S2: Statistical analysis of hypoxia-related mediators upregulated by SC144. OVCAR-8 cells were treated with 0.6 and 1.2 μM SC144 for 2 h, DMSO was used as vehicle control. Compared with control group: *$P < 0.05$, **$P < 0.01$.

Figure S3: Genes and perturbations positively related to SC144 treatment compared to control in OVCAR-8 cells. Bru-seq data was analysed by Connectivity Map L1000 Platform.

Figure S4: Genes and perturbations negatively related to SC144 treatment compared to control in OVCAR-8 cells. Bru-seq data was analysed by Connectivity Map L1000 Platform.

Figure S5: SC144 induces hypoxic stress, transiently up-regulation of HIF-1α and activation of NDRG1 signaling. (A) SC144 induces hypoxic stress. OVCAR-8 cells were treated with 1.2 μM SC144 for 2, 6, 12, 24 and 48 h, DMSO was used as vehicle control, DFO (100 μM, 24 h) was used as positive control. After treatments, cells were incubated with hypoxia green regent (Thermo Fisher Scientific, H20035) for 2 h, then cells were analysed using flow cytometry according to manufacturer's instructions. SC144 treatment significantly increases the hypoxia levels in different time points. (B) SC144 induces up-regulation of HIF-1α in a dose dependent manner as early as 2h. OVCAR-8 cells were treated with 1.2 and 2.4 μM SC144 for 2h. (C) SC144 induces transiently up-regulation of HIF-1α and up-regulation of NDRG1. ID 8 and LN-CaP cells were treated with 1.2 μM SC144 for 2, 6, 12, 24 and 48 h, DMSO was used as vehicle control. SC144 transiently increased the protein levels of ID8 and IN-CaP cells in 2 and 6 h's treatments, and then decreased in 12, 24 and 48 h's treatments. SC144 significantly increases the protein expression levels of NDRG1 on LN-CaP cells in an time-dependent manner. (D) Proteasome inhibition has no effect on the decrease of HIF-1α protein levels. OVCAR-8 and SK-OV-3 cells were treated with 10 μM MG-132 for 1, 2 and 4 h, then followed by SC144 treatment (1.2 μM, 24 h). DMSO was used as vehicle control and 2

h's SC144 treatment was used as positive control. Proteasome inhibition has no effect on SC144 induced decrease of HIF-1α protein levels in 24 h's treatment.

Figure S6: SC144 modulates NDRG1 and downstream signaling. (A) Protein expression of NDRG-1 and its select downstream signalling genes affected by SC144. Compared with DMSO control group: $*P<0.05$, $**P<0.01$. (B) SC144 decreases the protein expression levels of NDRG1 downstream factors, EGFR, Met and tissue factor (TF), in a time-dependent manner. OVCAR-8 and SK-OV-3 cells were treated with 1.2 μM SC144 for 2, 6, 12, 24 and 48 h, DMSO was used as vehicle control..

Figure S7: SC144 sensitizes ovarian cancer cells to chemotherapeutic agents. ID 8 cells were treated with the combinations of SC144 and chemotherapeutic agents including olaparib, carboplatin and cisplatin for 7 d, then, colonies were stained and imaged. Red lines indicate wells with significant synergistic effect from the combination treatment. Images are representative of 3 independent experiments.

Figure S8: SC144 exhibits metal chelation ability on iron, copper and zinc. The antiproliferative effects of SC144, CPX and DFO were rescued by Fe, Cu and Zn in a dose-dependent manner. SK-OV-3 cells were treated with SC144, CPX and DFO. FeCl3, CuSO4 and ZnCl2 were added together with the compounds at indicated concentrations Cell viabilities were determined using colony formation assays.

Figure S9: SC144 induces comparable cellular transcriptional profiling in both OBVAR-8 and LN CaP cells. (A) Common upregulated and downregulated genes by SC144 treatment between OVCAR-8 and LN CaP cells. Genes with a fold change of ±2 are considered in the analysis. (B) Common upregulated gene sets by SC144 treatment between OVCAR-8 and LN CaP cells.

Figure S10: Overlapped genes among top 25 upregulated genes by SC144 treatment between OVCAR-8 and LN CaP cells. Fold changes of the 9 overlapped genes from each cell lines are listed in the table. The gene map is from RefSeq Genes (UCSC genome browser, http://genome.ucsc.edu/).

Figure S11: Common compounds positively related to SC144 treatment in OVCAR-8 and LN CaP cells. Bru-seq data was analysed by Connectivity Map L1000 Platform. The compounds analysis was used a filter of median score>90. >edian scores of the 20 overlapped compounds from each cell lines are listed in the table.

Supplementary Figure S1

A

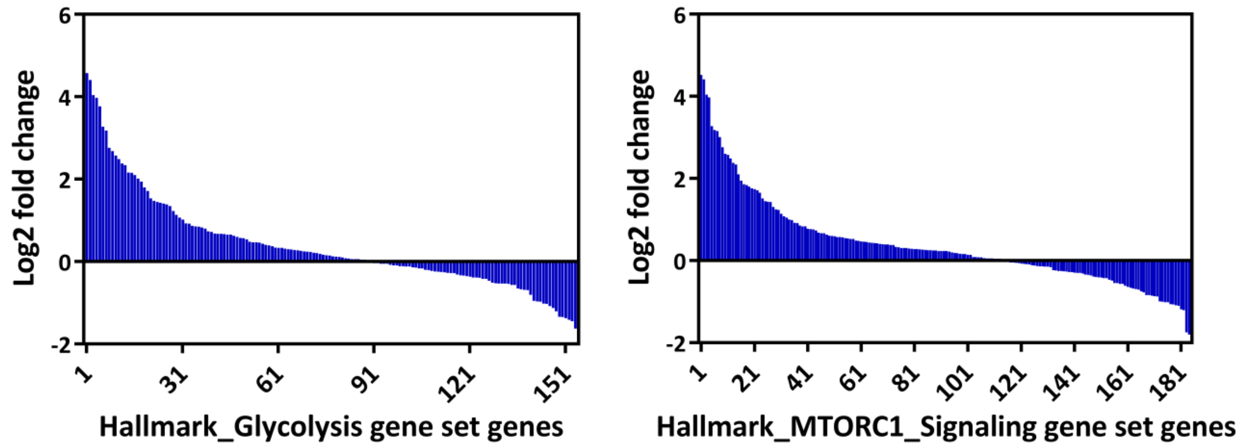

B

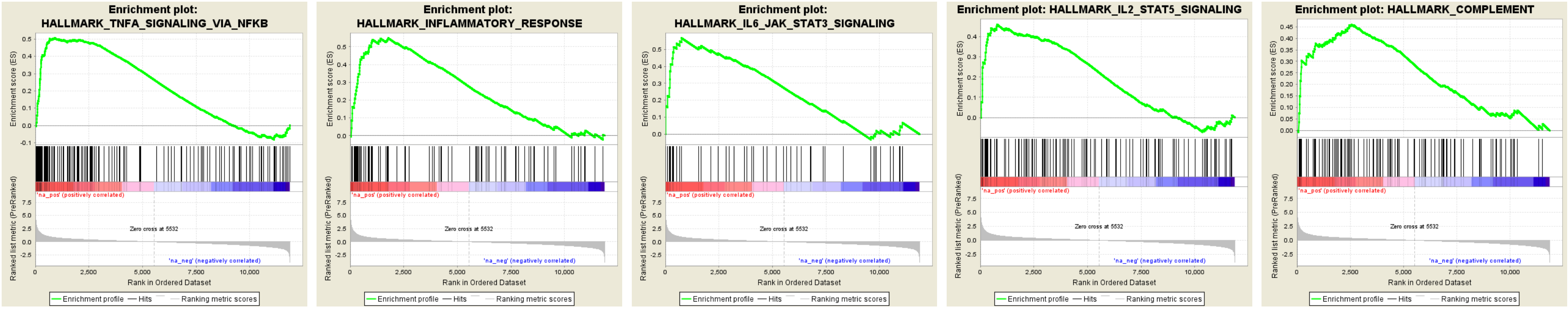

Supplementary Figure S2

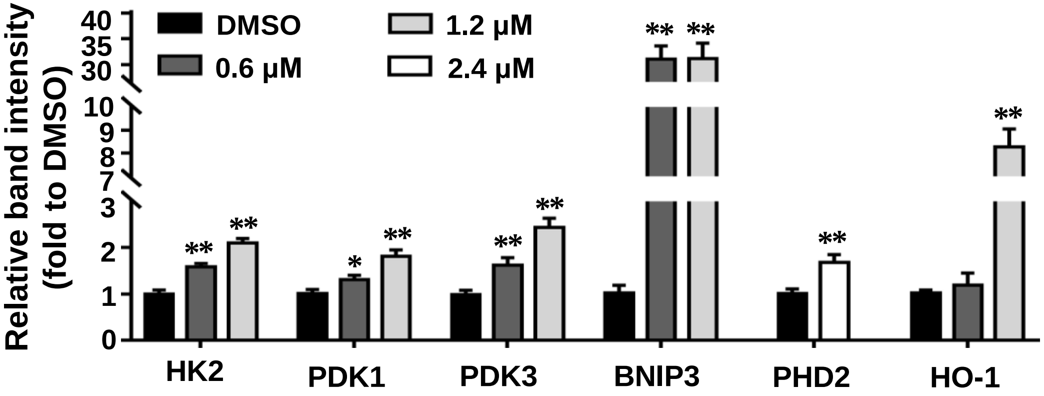

Supplementary Figure S3

cMap_gene and perturbation_OVCAR-8_Up

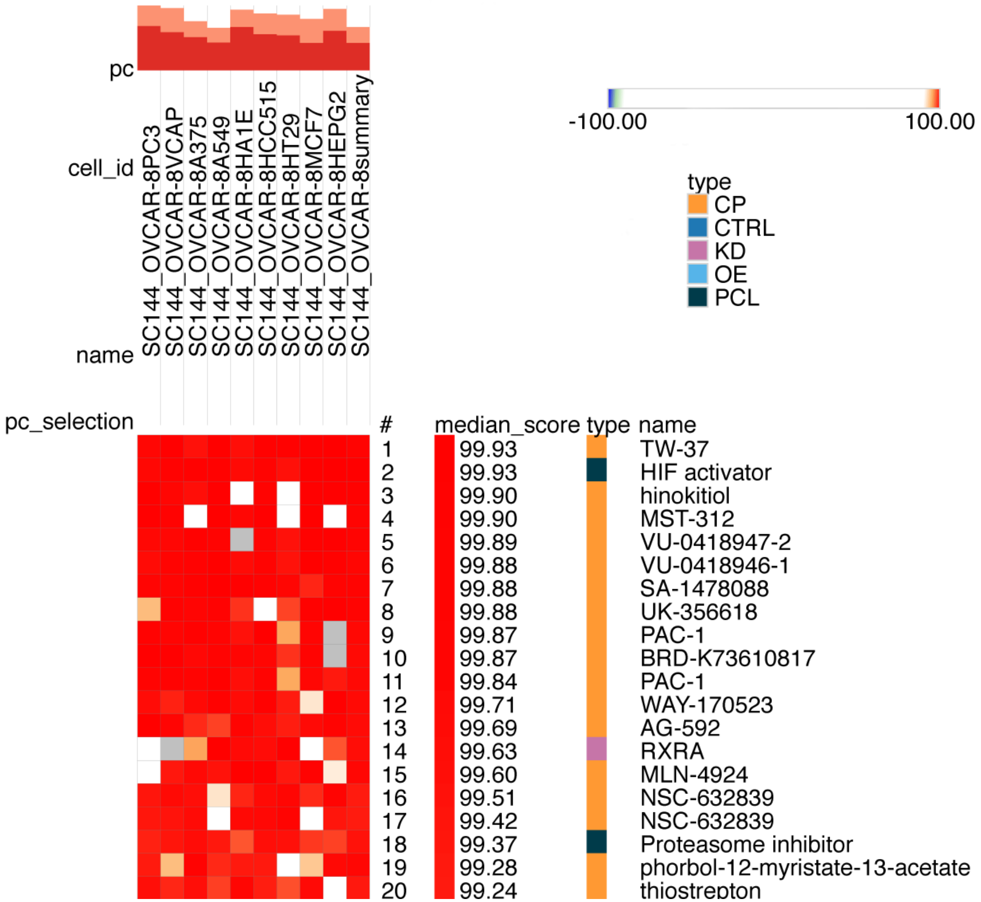

#	median_score	type	name	description
1	99.93		TW-37	BCL inhibitor, MCL1 inhibitor
2	99.93		HIF activator	
3	99.90		hinokitiol	tyrosinase inhibitor
4	99.90		MST-312	telomerase inhibitor
5	99.89		VU-0418947-2	hypoxia inducible factor activator
6	99.88		VU-0418946-1	hypoxia inducible factor activator
7	99.88		SA-1478088	metalloproteinase inhibitor
8	99.88		UK-356618	metalloproteinase inhibitor
9	99.87		PAC-1	caspase activator
10	99.87		BRD-K73610817	apoptosis protein inhibitor
11	99.84		PAC-1	caspase activator
12	99.71		WAY-170523	metalloproteinase inhibitor
13	99.69		AG-592	tyrosine kinase inhibitor
14	99.63		RXRA	Retinoid X receptors, retinoid X receptor, alpha
15	99.60		MLN-4924	nedd activating enzyme inhibitor
16	99.51		NSC-632839	ubiquitin hydrolase inhibitor, ubiquitin isopeptidase inhibitor
17	99.42		NSC-632839	ubiquitin hydrolase inhibitor, ubiquitin isopeptidase inhibitor
18	99.37		Proteasome inhibitor	
19	99.28		phorbol-12-myristate-13-acetate	PKC activator, CD antagonist
20	99.24		thiostrepton	downregulates FOXM1 expression, FOXM1 expression inh

Supplementary Figure S4

cMap_gene and perturbation_OVCAR-8_Down

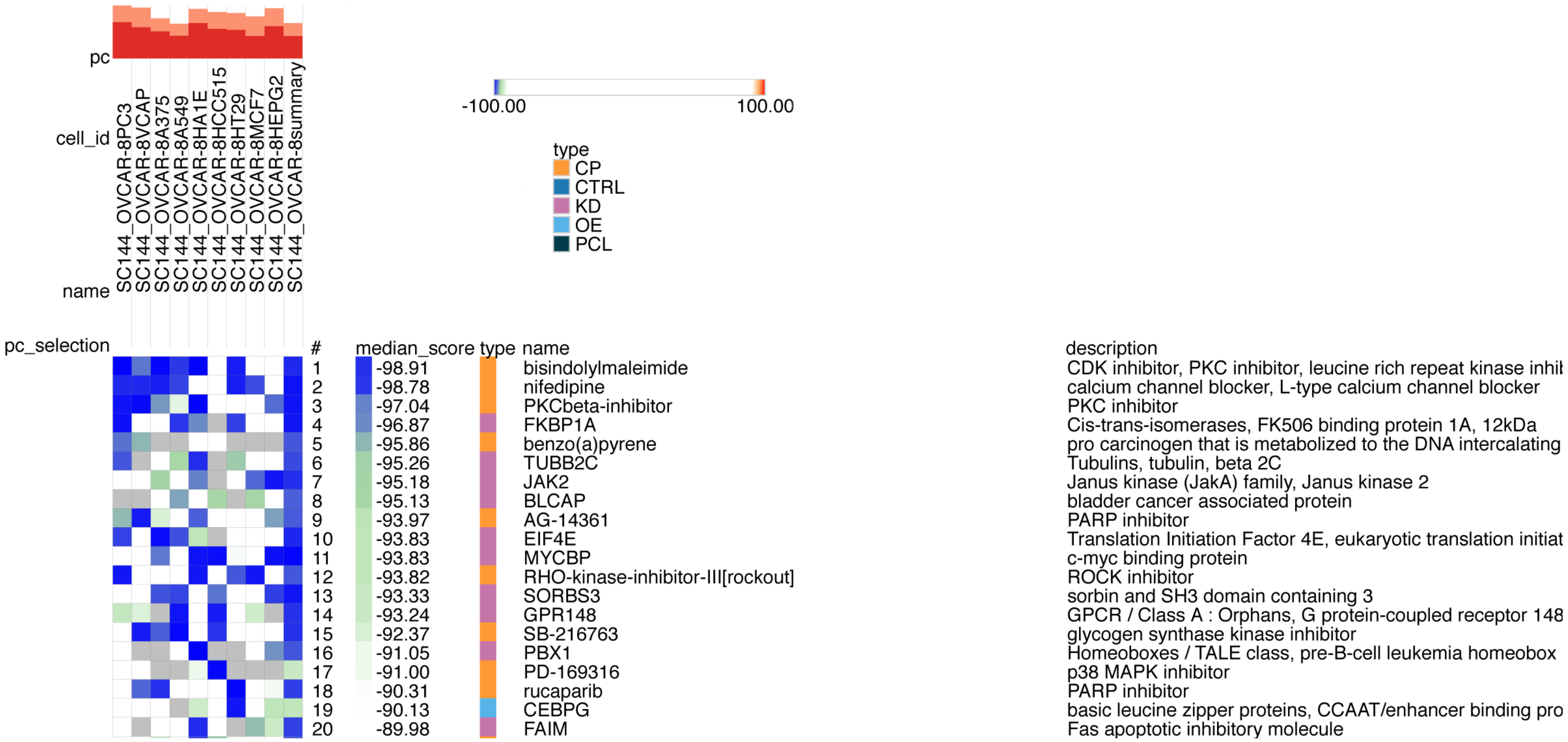

Supplementary Figure S5

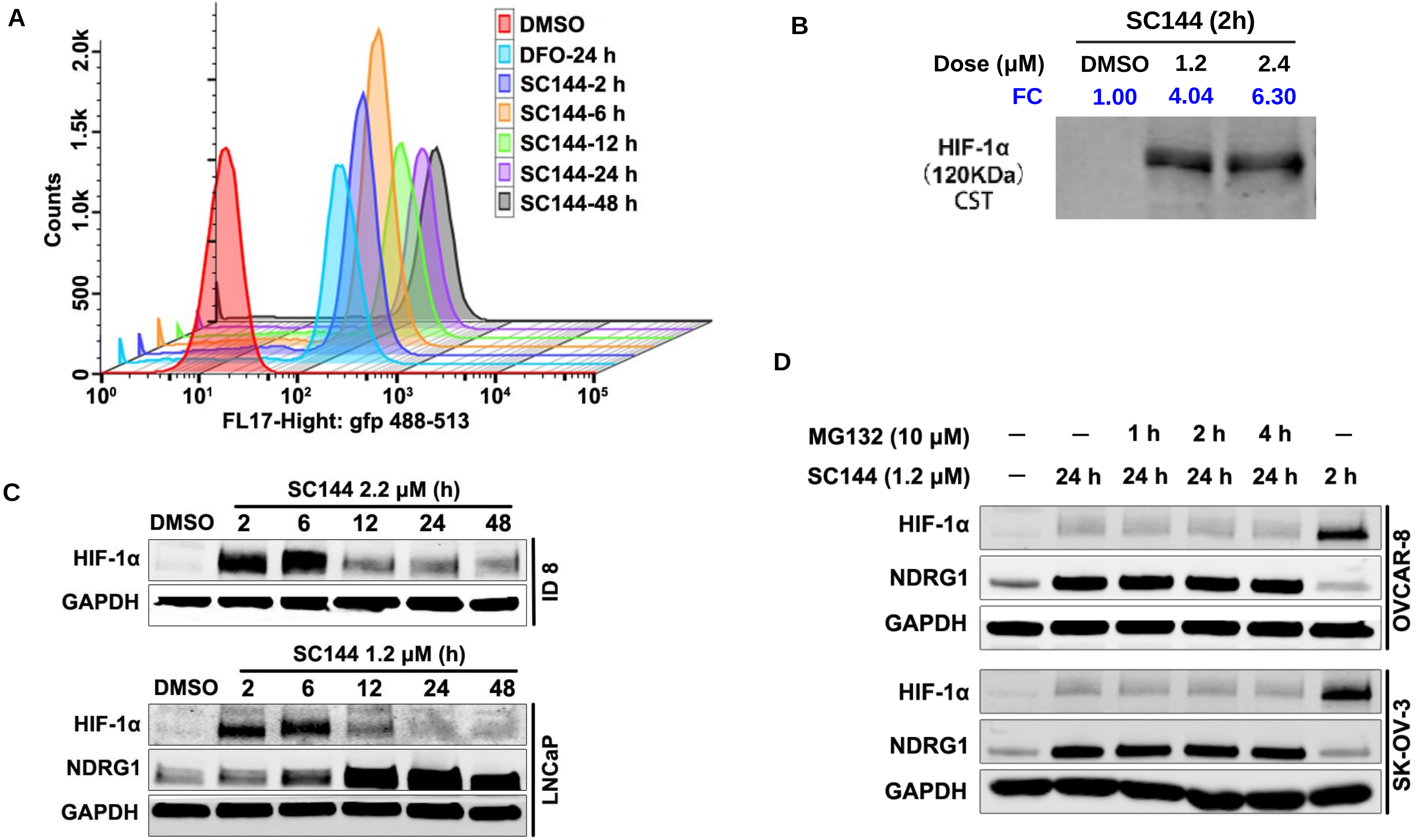

Supplementary Figure S6

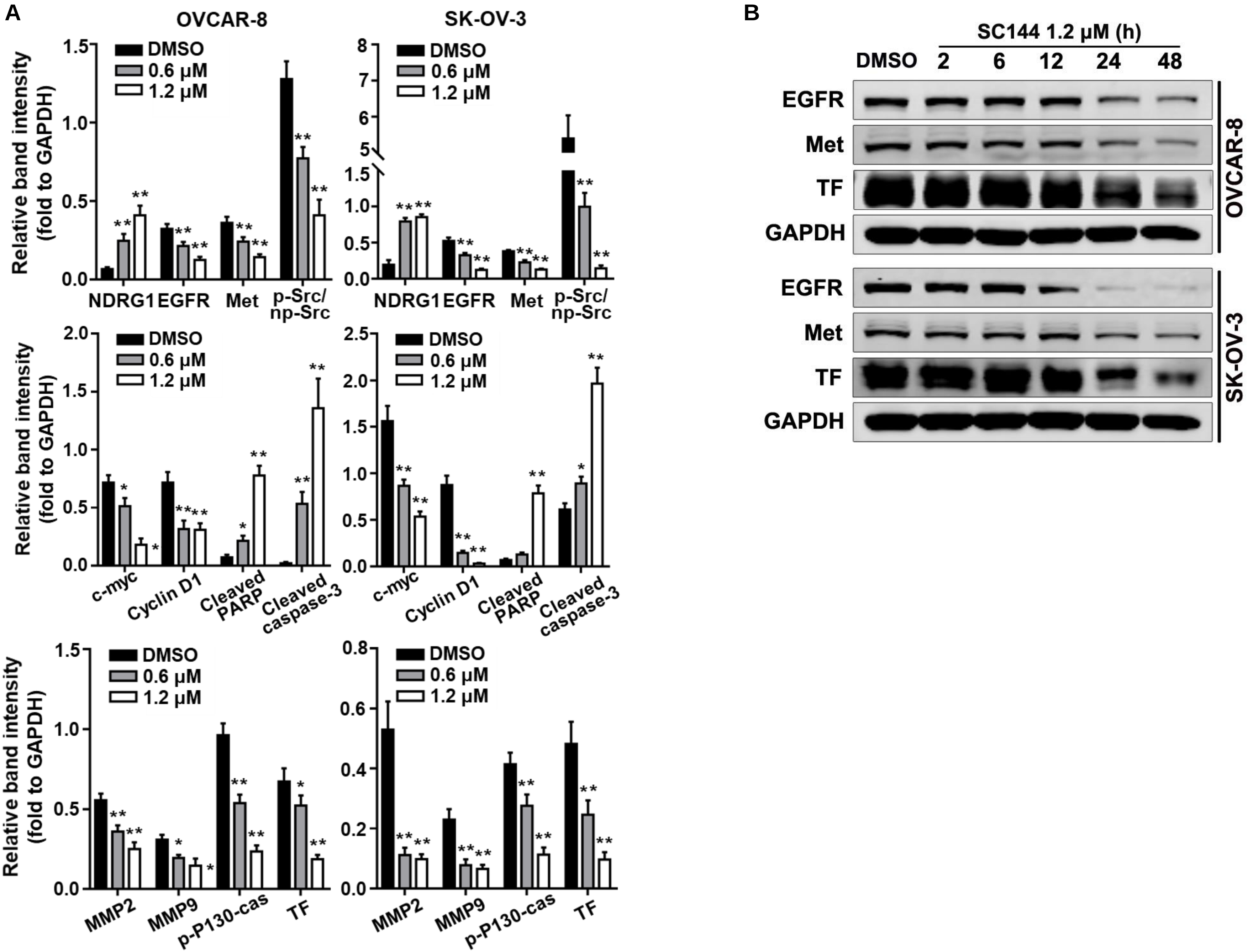

Supplementary Figure S7

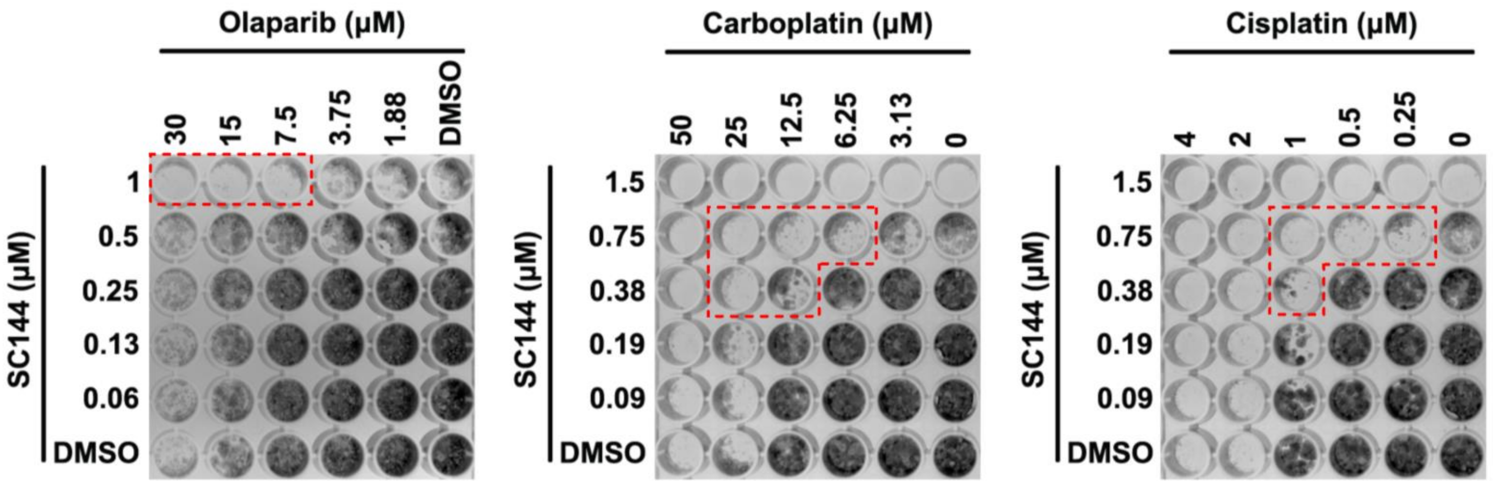

Supplementary Figure S8

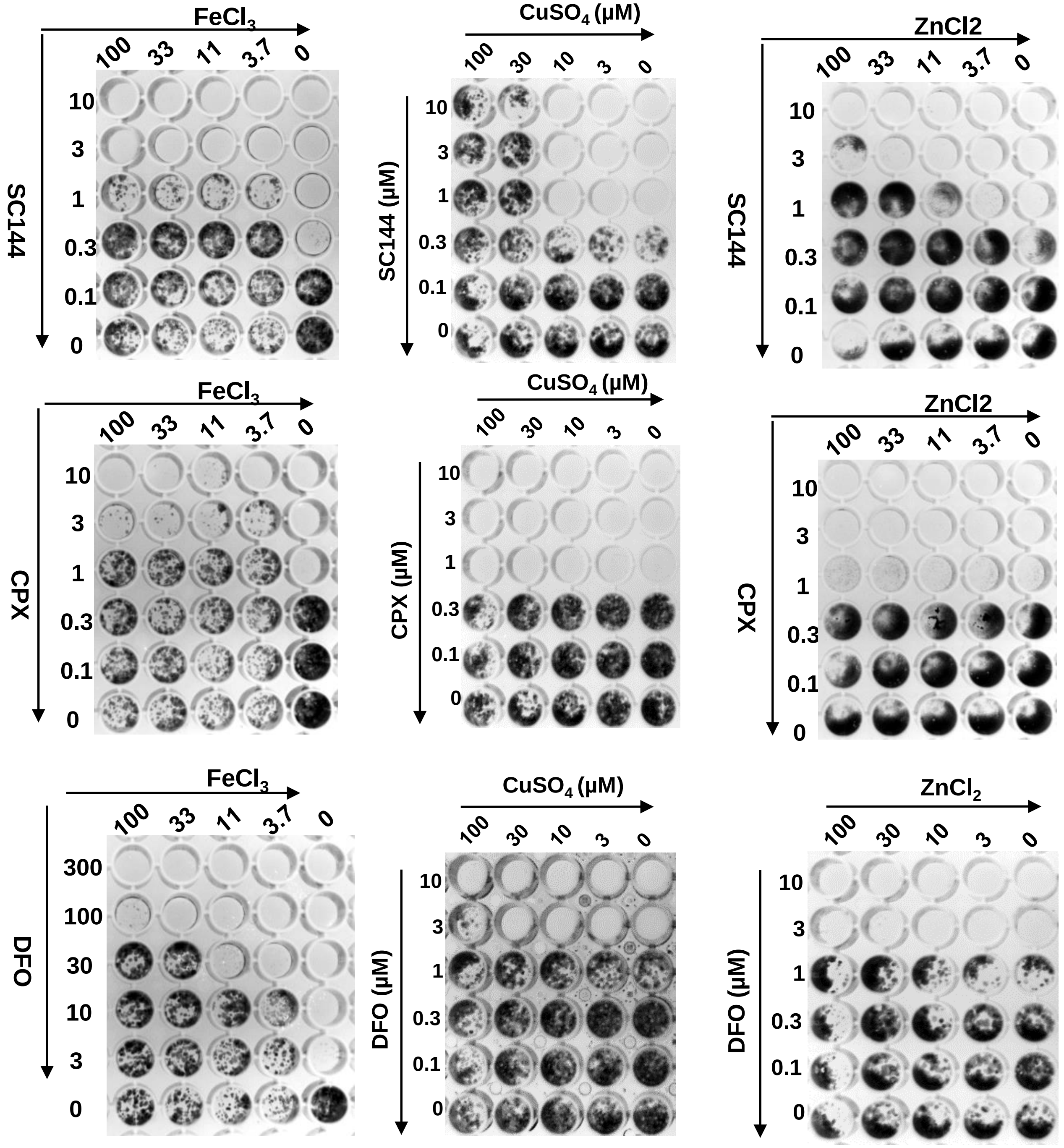

Supplementary Figure S9

A **SC144_Up regulated genes**

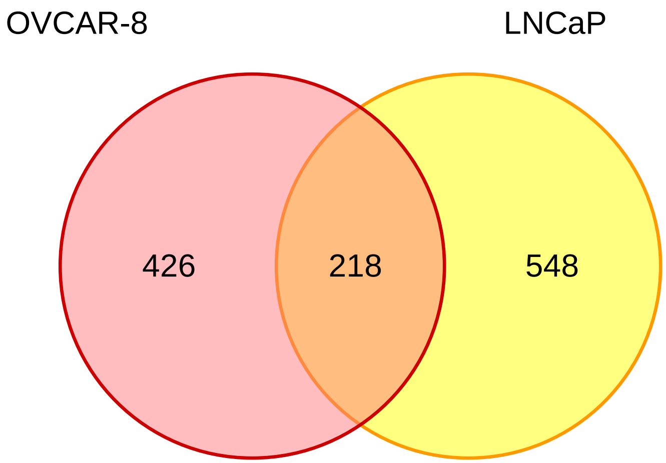

SC144_Down regulated genes

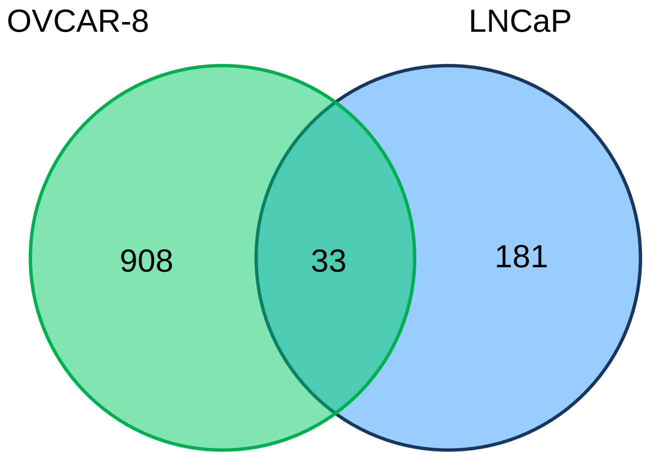

Cut off: ±2 fold change

B **SC144_Hallmark_Up regulated gene sets**

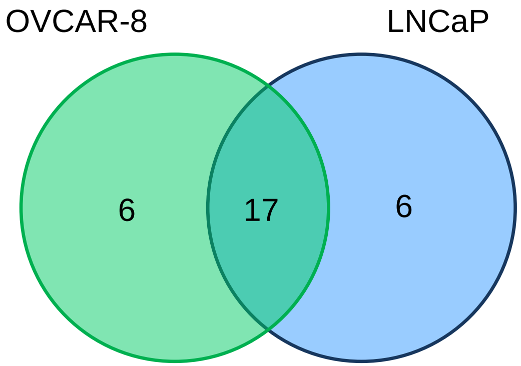

HYPOXIA
GLYCOLYSIS
MTORC1_SIGNALING
TNFA_SIGNALING_VIA_NFKB
INFLAMMATORY_RESPONSE
IL6_JAK_STAT3_SIGNALING
IL2_STAT5_SIGNALING
KRAS_SIGNALING_UP
HEME_METABOLISM
CHOLESTEROL_HOMEOSTASIS
P53_PATHWAY
ANDROGEN_RESPONSE
UV_RESPONSE_DN
HEDGEHOG_SIGNALING
ANGIOGENESIS
APOPTOSIS
ESTROGEN_RESPONSE_EARLY

Supplementary Figure S10

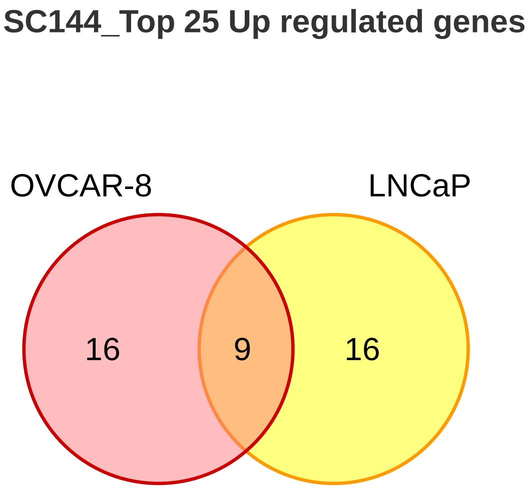

Gene	OVCAR-8	LNCaP
PPP1R3C	33.58	35.46
BNIP3	29.73	26.90
RIMKLA	22.21	67.35
PDK3	21.02	32.79
MT1X	17.86	27.30
C4orf47	16.95	75.84
RAB20	15.76	27.13
ANGPTL4	13.51	186.91
DTNA	11.76	22.61

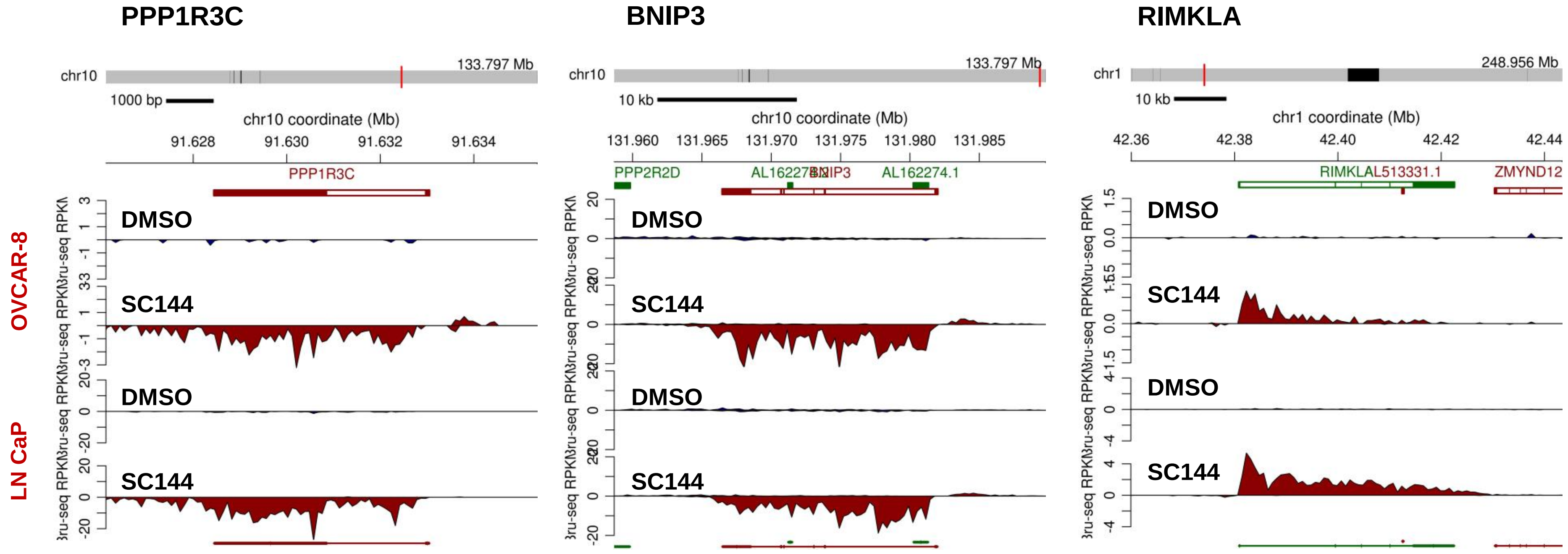

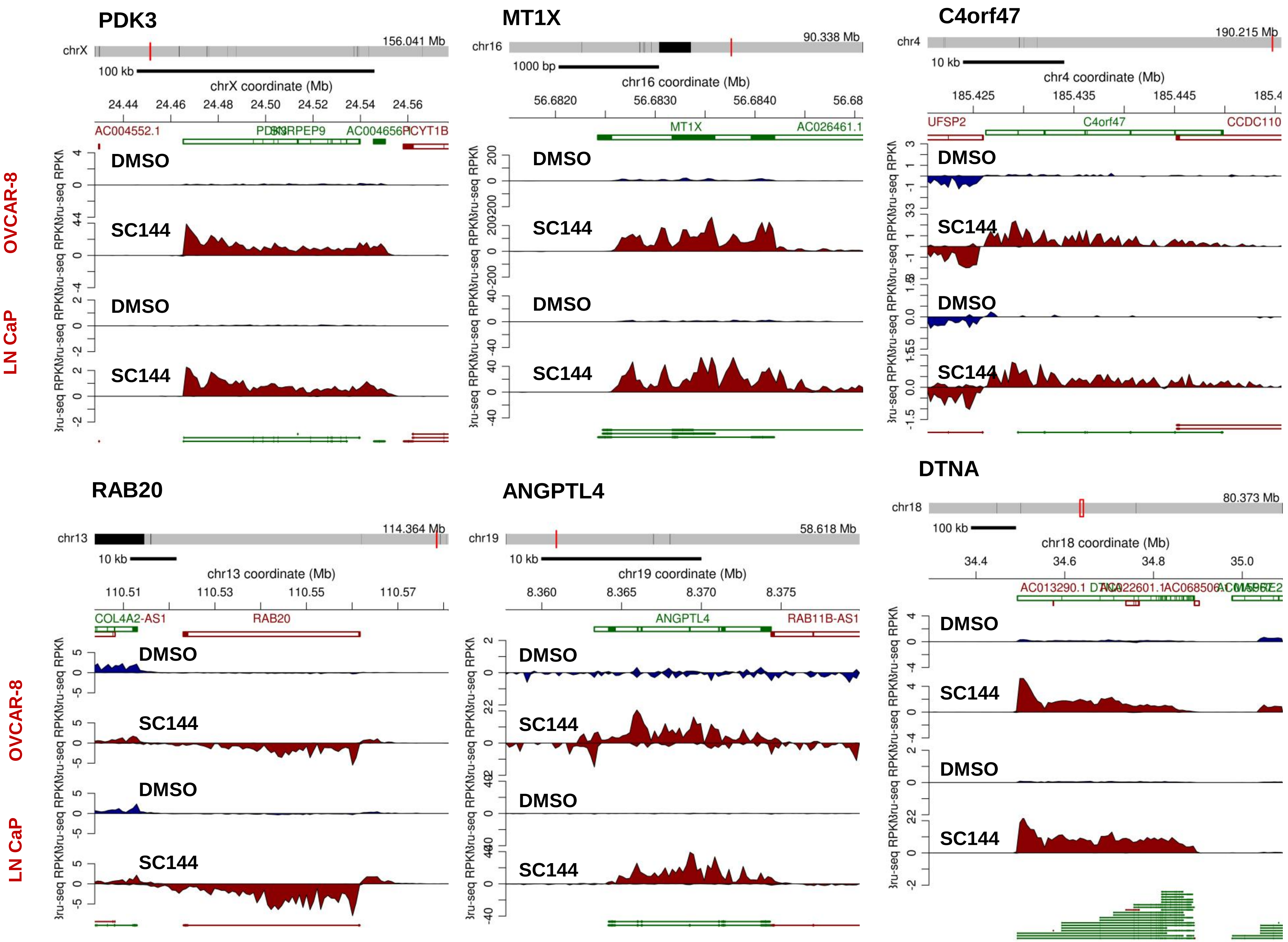
PDK3
chrX
156.041 Mb
100 kb
chrX coordinate (Mb)
24.44
24.46
24.48
24.50
24.52
24.54
24.56
AC004552.1
SNRPEP9
AC004656.1
CYT1B
MT1X
chr16
90.338 Mb
1000 bp
chr16 coordinate (Mb)
56.6820
56.6830
56.6840
MT1X
AC026461.1
C4orf47
chr4
190.215 Mb
10 kb
chr4 coordinate (Mb)
185.425
185.435
185.445
UFSP2
C4orf47
CCDC110
OVCAR-8
LN CaP
DMSO
SC144
DMSO
SC144
RAB20
chr13
114.364 Mb
10 kb
chr13 coordinate (Mb)
110.51
110.53
110.55
110.57
COL4A2-AS1
RAB20
ANGPTL4
chr19
58.618 Mb
10 kb
chr19 coordinate (Mb)
8.360
8.365
8.370
8.375
ANGPTL4
RAB11B-AS1
DTNA
chr18
80.373 Mb
100 kb
chr18 coordinate (Mb)
34.4
34.6
34.8
35.0
AC013290.1
OVCAR-8
LN CaP
DMSO
SC144
DMSO
SC144

Supplementary Figure S11

cMap_compound_positive

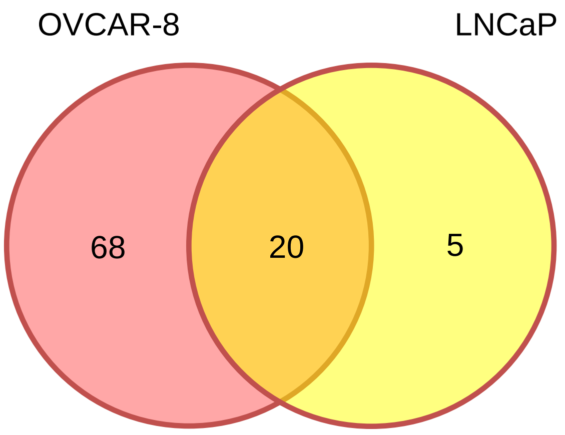

Filter: median score>90

	Name	Description	Median_score	
			OVCAR-8	LNCaP
1	TW-37	BCL inhibitor, MCL1 inhibitor	99.93	99.94
2	hinokitiol	tyrosinase inhibitor	99.9	99.94
3	MST-312	telomerase inhibitor	99.9	99.74
4	VU-0418947-2	hypoxia inducible factor activator	99.89	99.93
5	VU-0418946-1	hypoxia inducible factor activator	99.88	99.93
6	SA-1478088	metalloproteinase inhibitor	99.88	99.91
7	UK-356618	metalloproteinase inhibitor	99.88	99.29
8	BRD-K73610817	apoptosis protein inhibitor	99.87	99.87
9	PAC-1	caspase activator	99.87	99.94
10	WAY-170523	metalloproteinase inhibitor	99.71	95.82
11	AG-592	tyrosine kinase inhibitor	99.69	94.6
12	phorbol-12-myristate-13-acetate	PKC activator, CD antagonist	99.28	99.28
13	ingenol	PKC activator	97.71	97.69
14	prostratin	NFkB pathway activator, PKC activator	97.06	97.57
15	L-690488	nositol monophosphatase inhibitor	95.89	93.38
16	BIX-01294	DNA methyltransferase inhibitor	95.33	95.41
17	JTC-801	opioid receptor antagonist	94.73	92.21
18	lasalocid	ionophore antibiotic	94.49	94
19	gossypol	BCL inhibitor, MCL1 inhibitor	93.3	95.58
20	rhodomyrtoxin-b	cytotoxic, DNA intercalator	91.67	94.76

Table S1. Top 25 protein coding genes_Upregulated_SC144_OVCAR-8

**Gene ID**	**Description**	**Fold Change**
HOXA13	Homeobox A13	492.07
HMOX1	Heme Oxygenase 1	68.15
ARPP21	CAMP Regulated Phosphoprotein 21	36.94
PIK3IP1	Phosphoinositide-3-Kinase Interacting Protein 1	36.90
PPP1R3C	Protein Phosphatase 1 Regulatory Subunit 3C	33.58
BNIP3	BCL2 Interacting Protein 3	29.73
AKR1C3	Aldo-Keto Reductase Family 1 Member C3	24.73
FAM162A	Family With Sequence Similarity 162 Member A	23.68
PDK1	Pyruvate Dehydrogenase Kinase 1	22.81
RIMKLA	Ribosomal Modification Protein RimK Like Family Member A	22.21
SLC7A11	Solute Carrier Family 7 Member 11	21.19
EGLN1	Egl-9 Family Hypoxia Inducible Factor 1	21.06
PDK3	Pyruvate Dehydrogenase Kinase 3	21.02
NOL4	Nucleolar Protein 4	19.09
MT1X	Metallothionein 1X	17.86
C4orf47	Chromosome 4 Open Reading Frame 47	16.95
GLRX	Glutaredoxin	16.37
RAB20	RAB20, Member RAS Oncogene Family	15.76
DDIT4	DNA Damage Inducible Transcript 4	15.62
CXCL8	C-X-C Motif Chemokine Ligand 8	14.77
NDRG1	N-Myc Downstream Regulated 1	13.76
ANGPTL4	Angiopoietin Like 4	13.51
KDM7A	Lysine Demethylase 7A	12.17
DTNA	Dystrobrevin Alpha	11.76
MT1M	Metallothionein 1M	10.89

Table S2. Top 25 protein coding genes_Downregulated_SC144_OVCAR-8

**Gene ID**	**Description**	**Fold Change**
HIST1H1E	Histone Cluster 1 H1 Family Member E	0.07
HIST1H1B	Histone Cluster 1 H1 Family Member B	0.11
HIST1H2AL	Histone Cluster 1 H2A Family Member L	0.12
PLAU	Plasminogen Activator, Urokinase	0.12
HIST1H1D	Histone Cluster 1 H1 Family Member D	0.13
TXNIP	Thioredoxin Interacting Protein	0.14
HIST1H2AI	Histone Cluster 1 H2A Family Member I	0.15
HIST1H2BB	Histone Cluster 1 H2B Family Member B	0.16
HIST1H1C	Histone Cluster 1 H1 Family Member C	0.18
HIST1H2AM	Histone Cluster 1 H2A Family Member M	0.18
HIST1H2BK	Histone Cluster 1 H2B Family Member K	0.18
KRT80	Keratin 80	0.19
THBS1	Thrombospondin 1	0.19
TRMT61A	TRNA Methyltransferase 61A	0.20
HIST1H3C	Histone Cluster 1 H3 Family Member C	0.20
SSBP4	Single Stranded DNA Binding Protein 4	0.20
HIST1H2BM	Histone Cluster 1 H2B Family Member M	0.21
HIST2H2AC	Histone Cluster 2 H2A Family Member C	0.21
HIST1H3B	Histone Cluster 1 H3 Family Member B	0.21
HIST1H2AE	Histone Cluster 1 H2A Family Member E	0.21
TAGLN	Transgelin	0.22
OXTR	Oxytocin Receptor	0.22
GDF6	Growth Differentiation Factor 6	0.22
COX7A1	Cytochrome C Oxidase Subunit 7A1	0.23
ANKRD1	Ankyrin Repeat Domain 1	0.23

Table S3. Top 25 non-protein coding genes_Upregulated_SC144_OVCAR-8

**Gene ID**	**Biotype**	**Fold Change**
AC006372.1	lincRNA	274.20
EGLN1P1	transcribed_processed_pseudogene	196.47
AP002852.2	lincRNA	56.93
AC078883.1	antisense_RNA	43.61
SCAND2P	transcribed_unprocessed_pseudogene	39.80
AC140912.1	lincRNA	38.00
NMRAL2P	transcribed_unprocessed_pseudogene	32.09
LUCAT1	lincRNA	23.80
LINC00484	lincRNA	23.02
HLA-V	transcribed_unprocessed_pseudogene	22.65
AC097504.1	processed_pseudogene	17.49
AC026461.1	processed_transcript	13.73
PIK3IP1-AS1	antisense_RNA	12.92
MIR210HG	lincRNA	12.70
LINC02561	lincRNA	11.88
AC107918.4	transcribed_unprocessed_pseudogene	9.29
AKR1C7P	transcribed_unprocessed_pseudogene	8.21
AC006372.3	lincRNA	8.09
ENO1-AS1	antisense_RNA	7.98
AL355472.3	antisense_RNA	7.85
AC048382.5	antisense_RNA	7.79
AC107021.2	sense_overlapping	7.55
JHDM1D-AS1	antisense_RNA	6.92
AC114811.2	antisense_RNA	6.25
HIF1A-AS2	lincRNA	6.18

Table S4. Top 25 non-protein coding genes_Downregulated_SC144_OVCAR-8

**Gene ID**	**Biotype**	**Fold Change**
AC037198.2	sense_intronic	0.16
ELFN2	sense_overlapping	0.18
SNORA26	snoRNA	0.19
OR7H1P	unprocessed_pseudogene	0.22
AC124861.1	lincRNA	0.22
MIRLET7BHG	lincRNA	0.23
AP000892.3	TEC	0.24
OR7E19P	transcribed_unprocessed_pseudogene	0.24
AC123912.4	lincRNA	0.24
DANCR	processed_transcript	0.25
AC005363.1	transcribed_processed_pseudogene	0.25
MIR130B	processed_transcript	0.26
AC023137.1	lincRNA	0.26
SNORA71A	snoRNA	0.27
VPS9D1-AS1	antisense_RNA	0.27
PICSAR	lincRNA	0.28
SNORA24	snoRNA	0.28
AC245041.1	lincRNA	0.29
SLC12A9-AS1	antisense_RNA	0.29
RNASEH1-AS1	antisense_RNA	0.30
LINC00596	lincRNA	0.30
SNORA64	snoRNA	0.30
ADIRF-AS1	processed_transcript	0.31
SFTA3	processed_transcript	0.31
HMGB3P22	transcribed_processed_pseudogene	0.31

Table S5. Top 25 protein coding genes_Upregulated_ciclopirox_OVCAR-8

**Gene**	**Description**	**Fold Change**
HOXA13	Homeobox A13	301.01
PPP1R3C	Protein Phosphatase 1 Regulatory Subunit 3C	44.31
DDIT4	DNA Damage Inducible Transcript 4	43.41
BNIP3	BCL2 Interacting Protein 3	28.70
FAM162A	Family With Sequence Similarity 162 Member A	28.34
ANGPTL4	Angiopoietin Like 4	23.28
NDRG1	N-Myc Downstream Regulated 1	22.83
PDK1	Pyruvate Dehydrogenase Kinase 1	22.82
RIMKLA	Ribosomal Modification Protein RimK Like Family Member A	20.29
SPRY1	Sprouty RTK Signaling Antagonist 1	19.86
C4orf47	Chromosome 4 Open Reading Frame 47	16.81
PIK3IP1	Phosphoinositide-3-Kinase Interacting Protein 1	16.56
MT1X	Metallothionein 1X	16.04
HK2	Hexokinase 2	15.67
RAB20	RAB20, Member RAS Oncogene Family	15.65
CXCR4	C-X-C Motif Chemokine Receptor 4	13.33
EGLN1	Egl-9 Family Hypoxia Inducible Factor 1	12.75
BNIP3L	BCL2 Interacting Protein 3 Like	12.66
HMOX1	Heme Oxygenase 1	11.98
GLRX	Glutaredoxin	11.41
PFKFB4	6-Phosphofructo-2-Kinase/Fructose-2,6-Biphosphatase 4	10.85
NOL4	Nucleolar Protein 4	10.80
MT1M	Metallothionein 1M	10.55
CLEC2B	C-Type Lectin Domain Family 2 Member B	9.89
RNF122	Ring Finger Protein 122	9.87

Table S6. Top 25 protein coding genes_Downregulated_ciclopirox_OVCAR-8

**Gene**	**Description**	**Fold Change**
PLAU	Plasminogen Activator, Urokinase	0.092
TXNIP	Thioredoxin Interacting Protein	0.22
THBS1	Thrombospondin 1	0.23
NAPRT	Nicotinate Phosphoribosyltransferase	0.26
WDR73	WD Repeat Domain 73	0.29
HIST1H1E	Histone Cluster 1 H1 Family Member E	0.29
TRMT61A	TRNA Methyltransferase 61A	0.29
CXXC1	CXXC Finger Protein 1	0.31
SUSD3	Sushi Domain Containing 3	0.31
LPCAT4	Lysophosphatidylcholine Acyltransferase 4	0.31
EDN1	Endothelin 1	0.33
GTPBP3	GTP Binding Protein 3, Mitochondrial	0.33
RRP9	Ribosomal RNA Processing 9, U3 Small Nucleolar RNA Binding Protein	0.34
DUS1L	Dihydrouridine Synthase 1 Like	0.34
RHOT2	Ras Homolog Family Member T2	0.35
RRS1	Ribosome Biogenesis Regulator Homolog	0.36
ZNF512B	Zinc Finger Protein 512B	0.36
BOP1	Block Of Proliferation 1	0.37
INCENP	Inner Centromere Protein	0.37
FGF18	Fibroblast Growth Factor 18	0.37
CHTF18	Chromosome Transmission Fidelity Factor 18	0.37
ID3	Inhibitor Of DNA Binding 3, HLH Protein	0.37
RGS3	Regulator Of G Protein Signaling 3	0.37
TTLL12	Tubulin Tyrosine Ligase Like 12	0.37
CAPN10	Calpain 10	0.37

Table S7. Top 25 non-protein coding genes_Upregulated_ciclopirox_OVCAR-8

Gene	Biotype	Fold Change
AC006372.1	lincRNA	238.24
EGLN1P1	transcribed_processed_pseudogene	176.00
AC078883.1	antisense_RNA	40.23
LINC00484	lincRNA	33.36
SCAND2P	transcribed_unprocessed_pseudogene	32.04
AC140912.1	lincRNA	31.72
AP002852.2	lincRNA	27.50
HLA-V	transcribed_unprocessed_pseudogene	26.62
MIR210HG	lincRNA	21.80
AC097504.1	processed_pseudogene	19.63
HIF1A-AS2	lincRNA	12.95
AC026461.1	processed_transcript	12.04
SDAD1P1	transcribed_processed_pseudogene	11.15
NMRAL2P	transcribed_unprocessed_pseudogene	10.17
AC097534.2	antisense_RNA	9.92
AC107918.4	transcribed_unprocessed_pseudogene	9.03
AL355472.3	antisense_RNA	8.75
ENO1-AS1	antisense_RNA	8.27
AC107021.2	sense_overlapping	7.92
AP000695.1	antisense_RNA	7.82
DARS-AS1	antisense_RNA	7.60
LUCAT1	lincRNA	7.55
AC022400.8	TEC	7.49
LINC01291	lincRNA	7.42
AC048382.5	antisense_RNA	6.93

Table S8. Top 25 non-protein coding genes_Downregulated_ciclopirox_OVCAR-8

**Gene**	**Biotype**	**Fold Change**
AC037198.2	sense_intronic	0.19
OR7H1P	unprocessed_pseudogene	0.30
VPS9D1-AS1	antisense_RNA	0.30
MIR600HG	sense_intronic	0.34
SNORA71A	snoRNA	0.36
AC002558.1	processed_pseudogene	0.36
AC130462.1	processed_transcript	0.37
AC027307.3	lincRNA	0.37
TMEM147-AS1	antisense_RNA	0.39
FAM86C2P	transcribed_unprocessed_pseudogene	0.39
FAM222A-AS1	antisense_RNA	0.40
AL356356.1	antisense_RNA	0.40
AC005363.1	transcribed_processed_pseudogene	0.40
MIRLET7BHG	lincRNA	0.41
SLC12A9-AS1	antisense_RNA	0.41
DNM1P38	unprocessed_pseudogene	0.41
ADIRF-AS1	processed_transcript	0.42
SNORD83A	snoRNA	0.42
SNORA26	snoRNA	0.42
KNOP1P4	processed_pseudogene	0.43
SP2-AS1	antisense_RNA	0.43
SNORA64	snoRNA	0.43
AL391095.3	lincRNA	0.44
TYRO3P	processed_pseudogene	0.44
SNORA21	snoRNA	0.45

Table S9. Top 25 protein coding genes_Upregulated_SC144_LN CaP

**Gene ID**	**Description**	**Fold Change**
ANGPTL4	Angiopoietin Like 4	186.91
STC1	Stanniocalcin 1	116.57
CA9	Carbonic Anhydrase 9	100.84
EGLN3	Egl-9 Family Hypoxia Inducible Factor 3	78.91
C4orf47	Chromosome 4 Open Reading Frame 47	75.84
RIMKLA	Ribosomal Modification Protein RimK Like Family Member A	67.35
PPFIA4	PTPRF Interacting Protein Alpha 4	58.60
PFKFB4	6-Phosphofructo-2-Kinase/Fructose-2,6-Biphosphatase 4	57.78
ISM2	Isthmin 2	49.44
DOK3	Docking Protein 3	48.14
MTCP1	Mature T Cell Proliferation 1	44.62
PPP1R3C	Protein Phosphatase 1 Regulatory Subunit 3C	35.46
PDK3	Pyruvate Dehydrogenase Kinase 3	32.79
HK2	Hexokinase 2	32.73
HILPDA	Hypoxia Inducible Lipid Droplet Associated	31.35
SLC2A3	Solute Carrier Family 2 Member 3	30.81
STC2	Stanniocalcin 2	29.88
MT1X	Metallothionein 1X	27.30
RAB20	RAB20, Member RAS Oncogene Family	27.13
BNIP3	BCL2 Interacting Protein 3	26.90
IGFBP3	Insulin Like Growth Factor Binding Protein 3	26.74
DTNA	Dystrobrevin Alpha	22.61
GRIK4	Glutamate Ionotropic Receptor Kainate Type Subunit 4	22.40
ENO2	Enolase 2	21.69
CA12	Carbonic Anhydrase 12	21.07

Table S10. Top 25 protein coding genes_Downregulated_SC144_LN CaP

**Gene ID**	**Description**	**Fold Change**
TXNIP	Thioredoxin Interacting Protein	0.13
LHX4	LIM Homeobox 4	0.18
IGFBP2	Insulin Like Growth Factor Binding Protein 2	0.21
PELI1	Pellino E3 Ubiquitin Protein Ligase 1	0.21
MSX1	Msh Homeobox 1	0.24
MEGF9	Multiple EGF Like Domains 9	0.24
PER3	Period Circadian Regulator 3	0.24
C5orf67	Chromosome 5 Open Reading Frame 67	0.24
CYP24A1	Cytochrome P450 Family 24 Subfamily A Member 1	0.25
GGT5	Gamma-Glutamyltransferase 5	0.25
KCNK5	Potassium Two Pore Domain Channel Subfamily K Member 5	0.28
ANKDD1A	Ankyrin Repeat And Death Domain Containing 1A	0.29
ARL9	ADP Ribosylation Factor Like GTPase 9	0.30
WNT7B	Wnt Family Member 7B	0.30
CABLES1	Cdk5 And Abl Enzyme Substrate 1	0.31
AKR1B10	Aldo-Keto Reductase Family 1 Member B10	0.31
TMEM37	Transmembrane Protein 37	0.31
PPM1L	Protein Phosphatase, Mg2+/Mn2+ Dependent 1L	0.32
PLEKHH2	Pleckstrin Homology, MyTH4 And FERM Domain Containing H2	0.32
C4BPB	Complement Component 4 Binding Protein Beta	0.33
SLC16A5	Solute Carrier Family 16 Member 5	0.33
ZNF799	Zinc Finger Protein 799	0.33
TEF	TEF, PAR BZIP Transcription Factor	0.34
CHML	CHM Like, Rab Escort Protein 2	0.34
DIRAS2	DIRAS Family GTPase 2	0.34

Table S11. Top 25 non-protein coding genes_Upregulated_SC144_LN CaP

**Gene ID**	**Biotype**	**Fold Change**
TTC21B-AS1	processed_transcript	183.98
AC004656.1	sense_overlapping	79.35
AC097534.2	antisense_RNA	44.71
AC026461.1	processed_transcript	35.61
AC078883.1	antisense_RNA	28.34
EGLN1P1	transcribed_processed_pseudogene	23.24
SCAND2P	transcribed_unprocessed_pseudogene	22.48
LINC00887	lincRNA	19.45
HIF1A-AS2	lincRNA	17.10
AC010655.4	processed_transcript	17.08
SDAD1P1	transcribed_processed_pseudogene	15.76
AC022400.8	TEC	15.35
DARS-AS1	antisense_RNA	14.95
AL137918.1	TEC	14.67
AC136604.3	antisense_RNA	11.64
MIR210HG	lincRNA	11.57
HYMAI	non_coding	10.92
HLA-L	transcribed_unprocessed_pseudogene	10.06
AC011726.3	sense_intronic	9.93
LDHAP7	processed_pseudogene	9.68
AC011726.2	sense_intronic	8.70
NR2F2-AS1	antisense_RNA	8.58
AC016251.2	TEC	8.54
AC099795.2	processed_pseudogene	8.35
AC016251.1	TEC	8.27

Table S12. Top 25 non-protein coding genes_Downregulated_SC144_LN CaP

**Gene ID**	**Biotype**	**Fold Change**
AC105202.1	lincRNA	0.22
AL137129.1	processed_transcript	0.24
SFTA1P	lincRNA	0.26
PLCE1-AS1	antisense_RNA	0.27
AC091181.1	TEC	0.30
AC007684.1	sense_intronic	0.36
AC234772.2	lincRNA	0.36
AC018521.1	processed_transcript	0.36
LINC01750	lincRNA	0.37
AL603839.3	antisense_RNA	0.37
AC068888.1	antisense_RNA	0.40
AC103691.1	antisense_RNA	0.43
AP000941.1	antisense_RNA	0.43
AL356273.3	TEC	0.43
MYLK-AS1	antisense_RNA	0.44
AC008969.1	processed_transcript	0.44
AC107980.1	sense_intronic	0.45
LINC02331	lincRNA	0.46
AC009502.1	lincRNA	0.46
AC090833.1	lincRNA	0.47
AC006058.1	lincRNA	0.48
RN7SL2	misc_RNA	0.48
RPL24P8	processed_pseudogene	0.48
BTN2A3P	transcribed_unprocessed_pseudogene	0.49
LINC00472	lincRNA	0.49

Table S13. Gene sets positively correlated with SC144 OVCAR-8

NAME	SIZE	ES	NES	NOM p-val	FDR q-val	FWER p-val
HALLMARK_HYPOXIA	142	0.73	3.15	0	0	0
HALLMARK_GLYCOLYSIS	154	0.54	2.32	0	0	0
HALLMARK_MTORC1_SIGNALING	184	0.50	2.24	0	0	0
HALLMARK_TNFA_SIGNALING_VIA_NFKB	159	0.51	2.19	0	0	0
HALLMARK_INFLAMMATORY_RESPONSE	92	0.55	2.15	0	0	0
HALLMARK_IL6_JAK_STAT3_SIGNALING	46	0.57	1.97	0	0.003	0.009
HALLMARK_IL2_STAT5_SIGNALING	120	0.46	1.94	0	0.003	0.014
HALLMARK_COMPLEMENT	112	0.46	1.91	0	0.004	0.021
HALLMARK_REACTIVE_OXIGEN_SPECIES_PATHWAY	37	0.56	1.86	0.005	0.005	0.027
HALLMARK_KRAS_SIGNALING_UP	91	0.46	1.83	0	0.005	0.03
HALLMARK_PROTEIN_SECRETION	91	0.44	1.74	0	0.009	0.063
HALLMARK_HEME_METABOLISM	143	0.39	1.66	0	0.016	0.114
HALLMARK_CHOLESTEROL_HOMEOSTASIS	58	0.43	1.61	0.015	0.020	0.151
HALLMARK_P53_PATHWAY	150	0.37	1.60	0.005	0.022	0.175
HALLMARK_ANDROGEN_RESPONSE	84	0.40	1.58	0.005	0.023	0.192
HALLMARK_UV_RESPONSE_DN	123	0.37	1.57	0.004	0.023	0.202
HALLMARK_HEDGEHOG_SIGNALING	18	0.52	1.48	0.067	0.044	0.375
HALLMARK_XENOBIOTIC_METABOLISM	109	0.35	1.44	0.014	0.057	0.479
HALLMARK_BILE_ACID_METABOLISM	50	0.36	1.28	0.138	0.165	0.876
HALLMARK_ANGIOGENESIS	16	0.46	1.28	0.158	0.163	0.881
HALLMARK_APOPTOSIS	122	0.30	1.26	0.090	0.177	0.912
HALLMARK_INTERFERON_GAMMA_RESPONSE	119	0.30	1.25	0.104	0.181	0.928
HALLMARK_ESTROGEN_RESPONSE_EARLY	135	0.28	1.21	0.123	0.216	0.96

Table S14. Gene sets negatively correlated with SC144 OVCAR-8

NAME	SIZE	ES	NES	NOM p-val	FDR q-val	FWER p-val
HALLMARK_MYC_TARGETS_V2	56	-0.61	-2.21	0	0	0
HALLMARK_OXIDATIVE_PHOSPHORYLATION	179	-0.36	-1.60	0	0.038	0.134
HALLMARK_INTERFERON_ALPHA_RESPONSE	63	-0.37	-1.37	0.052	0.215	0.705
HALLMARK_DNA_REPAIR	124	-0.32	-1.35	0.048	0.187	0.755

Table S15. Gene sets positively correlated with ciclopirox OVCAR-8

NAME	SIZE	ES	NES	NOM p-val	FDR q-val	FWER p-val
HALLMARK_HYPOXIA	144	0.79	3.28	0	0	0
HALLMARK_GLYCOLYSIS	156	0.63	2.68	0	0	0
HALLMARK_MTORC1_SIGNALING	185	0.54	2.30	0	0	0
HALLMARK_TNFA_SIGNALING_VIA_NFKB	158	0.42	1.78	0	0.015	0.047
HALLMARK_IL2_STAT5_SIGNALING	117	0.44	1.73	0.002	0.018	0.069
HALLMARK_HEME_METABOLISM	142	0.39	1.61	0	0.050	0.208
HALLMARK_KRAS_SIGNALING_UP	91	0.41	1.59	0.004	0.049	0.230
HALLMARK_HEDGEHOG_SIGNALING	17	0.60	1.58	0.042	0.045	0.241
HALLMARK_INFLAMMATORY_RESPONSE	89	0.40	1.55	0.025	0.051	0.301
HALLMARK_PROTEIN_SECRETION	91	0.39	1.48	0.029	0.078	0.455
HALLMARK_REACTIVE_OXIGEN_SPECIES_PATHWAY	37	0.45	1.45	0.056	0.090	0.538
HALLMARK_P53_PATHWAY	150	0.34	1.41	0.027	0.115	0.655
HALLMARK_CHOLESTEROL_HOMEOSTASIS	58	0.39	1.39	0.065	0.119	0.689
HALLMARK_ANDROGEN_RESPONSE	84	0.35	1.33	0.071	0.159	0.808
HALLMARK_UV_RESPONSE_DN	121	0.32	1.26	0.115	0.238	0.926
HALLMARK_ADIPOGENESIS	145	0.30	1.26	0.072	0.227	0.930
HALLMARK_ESTROGEN_RESPONSE_EARLY	133	0.30	1.24	0.110	0.242	0.952

Table S16. Gene sets negatively correlated with ciclopirox OVCAR-8

NAME	SIZE	ES	NES	NOM p-val	FDR q-val	FWER p-val
HALLMARK_MYC_TARGETS_V2	56	-0.56	-1.98	0	0	0
HALLMARK_MITOTIC_SPINDLE	195	-0.36	-1.58	0	0.051	0.138
HALLMARK_G2M_CHECKPOINT	191	-0.35	-1.54	0.002	0.052	0.198
HALLMARK_E2F_TARGETS	195	-0.33	-1.47	0.002	0.078	0.359
HALLMARK_OXIDATIVE_PHOSPHORYLATION	179	-0.33	-1.46	0.008	0.072	0.401
HALLMARK_DNA_REPAIR	123	-0.35	-1.45	0.006	0.062	0.409
HALLMARK_APICAL_JUNCTION	114	-0.34	-1.41	0.018	0.074	0.520
HALLMARK_TGF_BETA_SIGNALING	48	-0.38	-1.32	0.091	0.131	0.796

Table S17. Gene sets positively correlated with SC144 LN CaP

NAME	SIZE	ES	NES	NOM p-val	FDR q-val	FWER p-val
HALLMARK_HYPOXIA	138	0.86	2.70	0	0	0
HALLMARK_GLYCOLYSIS	142	0.74	2.34	0	0	0
HALLMARK_TNFA_SIGNALING_VIA_NFKB	141	0.69	2.15	0	0	0
HALLMARK_MTORC1_SIGNALING	177	0.58	1.85	0	0.001	0.004
HALLMARK_EPITHELIAL_MESENCHYMAL_TRANSITION	115	0.55	1.71	0	0.011	0.057
HALLMARK_HEDGEHOG_SIGNALING	17	0.71	1.68	0.010	0.012	0.073
HALLMARK_KRAS_SIGNALING_UP	75	0.55	1.62	0.002	0.020	0.138
HALLMARK_MYOGENESIS	80	0.54	1.62	0.001	0.020	0.159
HALLMARK_INFLAMMATORY_RESPONSE	87	0.52	1.57	0.008	0.034	0.283
HALLMARK_IL6_JAK_STAT3_SIGNALING	42	0.56	1.54	0.020	0.040	0.342
HALLMARK_COAGULATION	49	0.54	1.52	0.019	0.047	0.417
HALLMARK_HEME_METABOLISM	131	0.48	1.50	0.006	0.050	0.454
HALLMARK_UV_RESPONSE_DN	109	0.49	1.50	0.007	0.047	0.458
HALLMARK_P53_PATHWAY	144	0.48	1.50	0.007	0.045	0.468
HALLMARK_IL2_STAT5_SIGNALING	112	0.48	1.46	0.010	0.061	0.607
HALLMARK_UNFOLDED_PROTEIN_RESPONSE	96	0.45	1.37	0.057	0.133	0.895
HALLMARK_TGF_BETA_SIGNALING	46	0.49	1.35	0.116	0.153	0.931
HALLMARK_ANGIOGENESIS	15	0.59	1.35	0.129	0.150	0.942
HALLMARK_ESTROGEN_RESPONSE_EARLY	133	0.42	1.33	0.048	0.161	0.954
HALLMARK_ANDROGEN_RESPONSE	80	0.43	1.30	0.102	0.192	0.982
HALLMARK_ALLOGRAFT_REJECTION	79	0.43	1.29	0.103	0.189	0.987
HALLMARK_APOPTOSIS	115	0.41	1.29	0.091	0.186	0.988
HALLMARK_CHOLESTEROL_HOMEOSTASIS	55	0.45	1.27	0.155	0.213	0.993

Table S18. Gene sets negatively correlated with SC144 LN CaP

NAME	SIZE	ES	NES	NOM p-val	FDR q-val	FWER p-val
HALLMARK_MYC_TARGETS_V2	51	-0.40	-1.58	0.005	0.103	0.071
HALLMARK_KRAS_SIGNALING_DN	37	-0.44	-1.54	0.031	0.062	0.083
HALLMARK_E2F_TARGETS	188	-0.28	-1.25	0.040	0.219	0.475

Table S19. cMap_Compound_SC144_OVCAR-8_Up

Name	Description	Target	Median_score
TW-37	BCL inhibitor, MCL1 inhibitor	BCL2, BCL2L1, MCL1	99.93
hinokitiol	tyrosinase inhibitor	TYR	99.9
MST-312	telomerase inhibitor	TERT	99.9
VU-0418947-2	hypoxia inducible factor activator		99.89
VU-0418946-1	hypoxia inducible factor activator	HIF1A	99.88
SA-1478088	metalloproteinase inhibitor	MMP12, MMP14, MMP2, MMP8, MMP9	99.88
UK-356618	metalloproteinase inhibitor	MMP3, MMP13, MMP14, MMP2, MMP9	99.88
BRD-K73610817	apoptosis protein inhibitor		99.87
PAC-1	caspase activator	CASP3	99.87
PAC-1	caspase activator	CASP3	99.84
WAY-170523	metalloproteinase inhibitor	MMP13	99.71
AG-592	tyrosine kinase inhibitor		99.69
MLN-4924	nedd activating enzyme inhibitor	NAE1, UBA3	99.6
NSC-632839	ubiquitin hydrolase inhibitor, ubiquitin isopeptidase inhibitor	USP2, USP7, SENP2, USP1	99.51
NSC-632839	ubiquitin hydrolase inhibitor, ubiquitin isopeptidase inhibitor	USP2, USP7, SENP2, USP1	99.42
phorbol-12-myristate-13-acetate	PKC activator, CD antagonist	CD4, KCNT2, PRKCA, TRPV4	99.28
thiostrepton	downregulates FOXM1 expression, FOXM1 expression inhibitor, protein synthesis inhibitor	FOXM1	99.24
disulfiram	aldehyde dehydrogenase inhibitor, DNA methyltransferase inhibitor, TRPA1 agonist	ALDH2, ALDH1A2, ALDH5A1, ALDH7A1, CYP2E1, DBH, DNMT1, TRPA1	99.24
MLN-2238	proteasome inhibitor	PSMB1	99.12
iodoacetic-acid	cysteine peptidase inhibitor		99.11
z-leu3-VS	PGPH inhibitor, proteasome inhibitor		99.1
15-delta-prostaglandin-j2	PPAR receptor agonist, FXR antagonist	PPARG, NR1H4	99.02
CA-074-Me	cathepsin inhibitor, antiamyloidogenic agent	CTSB	98.99
parthenolide	NFkB pathway inhibitor, adiponectin receptor agonist	ADIPOR2, IKBKB, RELA	98.9
MG-132	proteasome inhibitor	PSMB1	98.88

Table S20. cMap_Compound_SC144_OVCAR-8_Down

Name	Description	Target	Median_score
bisindolylmaleimide	CDK inhibitor, PKC inhibitor, leucine rich repeat kinase inhibitor	CCND1, CDK4, LRRK2, PDPK1, PIM1, PRKCA, PRKCB, PRKCI, PRKCZ	-98.91
nifedipine	calcium channel blocker, L-type calcium channel blocker	CACNA1C, CACNA1D, CACNA1S, CACNA2D1, CACNA1F, CACNA1H, CACNB2, CALM1, CYP3A5, GLRA1, GLRA3, GLRB, KCNA1, KCNA5, NR1I2, TRPM3	-98.78
PKCbeta-inhibitor	PKC inhibitor	PRKCB	-97.04
benzo(a)pyrene	pro carcinogen that is metabolized to the DNA intercalating agent benzo(a)pyrene diol epoxide		-95.86
AG-14361	PARP inhibitor	PARP1	-93.97
RHO-kinase-inhibitor-III[rockout]	ROCK inhibitor	IMPDH2, ROCK1	-93.82
SB-216763	glycogen synthase kinase inhibitor	GSK3B, CCNA2, CDK2, GSK3A	-92.37
PD-169316	p38 MAPK inhibitor	ALOX5	-91
rucaparib	PARP inhibitor	PARP1, PARP2	-90.31
curcumin	cyclooxygenase inhibitor, AP inhibitor, CCN expression inhibitor, DNA methyltransferase inhibitor, EGFR expression inhibitor, free radical scavenger, FtsZ inhibitor, glucose 6 phosphatase inhibitor, histone N-acetyltransferase inhibitor, HIV integrase inhibitor, lipoxygenase inhibitor, NFkB pathway inhibitor, tau aggregation inhibitor, unidentified pharmacological activity	PTGS1, PTGS2, APP, CA1, CA12, CA14, CA2, CA4, CA6, CA9, CCND1, CYP3A4, DNMT1, DNMT3B, EP300, G6PC, MAPT, MMP13, MMP9, NOS2, XDH	-89.71
kenpaullone	CDK inhibitor, glycogen synthase kinase inhibitor, src inhibitor	GSK3B, CDK1, CDK5, CCNB1, CDK2, LCK	-89.69
enzastaurin	PKC inhibitor, AKT inhibitor, angiogenesis inhibitor, apoptosis stimulant, PI3K inhibitor	PRKCB, AKT1, GSK3B, PRKCA, PRKCD, PRKCG	-88.46
OM-137	Aurora kinase inhibitor		-85.48
YK-4279	apoptosis stimulant, binding of RNA helicase A to the transcription factor EWS-FLI1 inhibitor, EWS-FLI1 inhibitor	DHX9, EWSR1, FLI1	-85.27
enalaprilat	angiotensin converting enzyme inhibitor	ACE	-85.01
fatostatin	sterol regulatory element binding protein (SREBP) inhibitor	SREBF1, SREBF2	-84.48

SB-218078	CHK inhibitor, PKC inhibitor	CHEK1	-83.88
SB-269970	serotonin receptor antagonist	HTR7	-81.11
indirubin	CDK inhibitor, glycogen synthase kinase inhibitor, cyclin-dependent kinase inhibitor, PKC inhibitor	CDK1, CDK5, CCNE1, CDK2, CDK4, CDK5R1, CDK9, GSK3A, GSK3B, LCK, LRRK1, LRRK2	-80.55
nifedipine	calcium channel blocker, L-type calcium channel blocker	CACNA1C, CACNA1D, CACNA1S, CACNA2D1, CACNA1F, CACNA1H, CACNB2, CALM1, CYP3A5, GLRA1, GLRA3, GLRB, KCNA1, KCNA5, NR1I2, TRPM3	-80.32
etomoxir	carnitine palmitoyltransferase inhibitor, carnitine O-palmitoyltransferase inhibitor, fatty acid oxidation inhibitor	CPT1A, CPT1B	-80.04

Table S21. cMap_Compound_ciclopirox_OVCAR-8_Up

**Name**	**Description**	**Target**	**Median_score**
VU-0418946-1	hypoxia inducible factor activator	HIF1A	99.98
SA-1478088	metalloproteinase inhibitor	MMP12, MMP14, MMP2, MMP8, MMP9	99.97
TW-37	BCL inhibitor, MCL1 inhibitor	BCL2, BCL2L1, MCL1	99.97
hinokitiol	tyrosinase inhibitor	TYR	99.97
BRD-K73610817	apoptosis protein inhibitor		99.96
VU-0418947-2	hypoxia inducible factor activator		99.96
PAC-1	caspase activator	CASP3	99.94
MST-312	telomerase inhibitor	TERT	99.94
PAC-1	caspase activator	CASP3	99.93
WAY-170523	metalloproteinase inhibitor	MMP13	99.92
BAPTA-AM	potassium channel blocker	KCNA3, KCNA5, KCNH2	99.88
UK-356618	metalloproteinase inhibitor	MMP3, MMP13, MMP14, MMP2, MMP9	99.74
AG-592	tyrosine kinase inhibitor		99.69
MLN-4924	nedd activating enzyme inhibitor	NAE1, UBA3	99.41
BRD-A54632525	lipoxygenase inhibitor	ALOX12	99.26
NSC-632839	ubiquitin hydrolase inhibitor, ubiquitin isopeptidase inhibitor	USP2, USP7, SENP2, USP1	99.21
NSC-3852	HDAC inhibitor	HDAC1	99.08
linifanib	PDGFR tyrosine kinase receptor inhibitor, VEGFR inhibitor, angiogenesis inhibitor, colony stimulating factor receptor antagonist, colony stimulating factor receptor inhibitor, FLT3 inhibitor, macrophage colony stimulating factor antagonist, MAP kinase inhibitor, PARP inhibitor, receptor tyrosine kinase inhibitor, STAT inhibitor, VEGFR antagonist	CSF1R, KDR, PDGFRB, FLT1, FLT3, FLT4, CSF1, KIT, PDGFRA, RET, TEK	98.89

BX-912	AKT inhibitor, phosphoinositide dependent kinase inhibitor, pyruvate dehydrogenase kinase inhibitor	PDPK1, AKT2, CDK2, CHEK1, GSK3B, KDR, PDK1	98.85
gossypol	BCL inhibitor, MCL1 inhibitor, 11-beta hydroxysteroid dehydrogenase inhibitor, growth factor receptor modulator, lipid peroxidase inhibitor	BCL2, BCL2L1, MCL1, BCL2L2, CTGF, EGF	98.74
BIX-01294	DNA methyltransferase inhibitor, histone lyinse methyltransferase inhibitor, histone lysine methyltransferase inhibitor	EHMT1, EHMT2	98.72
NSC-632839	ubiquitin hydrolase inhibitor, ubiquitin isopeptidase inhibitor	USP2, USP7, SENP2, USP1	98.57
disulfiram	aldehyde dehydrogenase inhibitor, DNA methyltransferase inhibitor, TRPA1 agonist	ALDH2, ALDH1A2, ALDH5A1, ALDH7A1, CYP2E1, DBH, DNMT1, TRPA1	98.52
BMY-45778	IP1 prostacyclin receptor agonist	PTGIR	98.5
heliomycin	antibiotic, bacterial RNA synthesis inhibitor		98.49
chloroxine	opioid receptor inhibitor	OPRK1	98.43
thiostrepton	downregulates FOXM1 expression, FOXM1 expression inhibitor, protein synthesis inhibitor	FOXM1	98.23
puromycin	adenosine receptor agonist, protein synthesis inhibitor	NHP2L1, RPL10L, RPL11, RPL13A, RPL15, RPL19, RPL23, RPL23A, RPL26L1, RPL3, RPL37, RPL8, RSL24D1	98.02
APHA-compound-8	HDAC inhibitor	HDAC8	98

Table S22. cMap_Compound_ciclopirox_OVCAR-8_Down

Name	Description	Target	Median_score
SB-216763	glycogen synthase kinase inhibitor	GSK3B, CCNA2, CDK2, GSK3A	-99.26
PKCbeta-inhibitor	PKC inhibitor	PRKCB	-97.35
YK-4279	apoptosis stimulant, binding of RNA helicase A to the transcription factor EWS-FLI1 inhibitor, EWS-FLI1 inhibitor	DHX9, EWSR1, FLI1	-96.56
PD-169316	p38 MAPK inhibitor	ALOX5	-95.9
benzo(a)pyrene	pro carcinogen that is metabolized to the DNA intercalating agent benzo(a)pyrene diol epoxide		-95.36
RHO-kinase-inhibitor-III[rockout]	ROCK inhibitor	IMPDH2, ROCK1	-94.83
rucaparib	PARP inhibitor	PARP1, PARP2	-94.51
SB-218078	CHK inhibitor, PKC inhibitor	CHEK1	-93.35
bisindolylmaleimide	CDK inhibitor, PKC inhibitor, leucine rich repeat kinase inhibitor	CCND1, CDK4, LRRK2, PDPK1, PIM1, PRKCA, PRKCB, PRKCI, PRKCZ	-92.95
epothilone	inhibition of microtubulefunction, microtubule stabilizing agent, microtubule stimulant, tubulin inhibitor	TUBA1A, TUBA1B, TUBA1C, TUBA3C, TUBA4A, TUBA8, TUBB, TUBB1, TUBB3, TUBB4A, TUBB4B	-91.39
ABT-751	tubulin inhibitor, dihydropteroate synthase inhibitor, microtubule inhibitor, PABA antagonist, tubulin polymerisation inhibitor	TUBB	-91.01

Table S23. cMap_Compound_SC144_LN CaP_Up

Name	Description	Target	Median_score
hinokitiol	tyrosinase inhibitor	TYR	99.94
PAC-1	caspase activator	CASP3	99.94
TW-37	BCL inhibitor, MCL1 inhibitor	BCL2, BCL2L1, MCL1	99.94
VU-0418947-2	hypoxia inducible factor activator		99.93
VU-0418946-1	hypoxia inducible factor activator	HIF1A	99.93
SA-1478088	metalloproteinase inhibitor	MMP12, MMP14, MMP2, MMP8, MMP9	99.91
PAC-1	caspase activator	CASP3	99.91
BRD-K73610817	apoptosis protein inhibitor		99.87
MST-312	telomerase inhibitor	TERT	99.74
UK-356618	metalloproteinase inhibitor	MMP3, MMP13, MMP14, MMP2, MMP9	99.29
ingenol	PKC activator	PRKCD, PRKCE	97.69
prostratin	NFkB pathway activator, PKC activator	PRKCA, PRKCB, PRKCD, PRKCE, PRKCG, PRKCH, PRKCQ	97.57
rhodomyrtoxin	cytotoxic, DNA intercalator		97.48
WAY-170523	metalloproteinase inhibitor	MMP13	95.82
gossypol	BCL inhibitor, MCL1 inhibitor, 11-beta hydroxysteroid dehydrogenase inhibitor, growth factor receptor modulator, lipid peroxidase inhibitor	BCL2, BCL2L1, MCL1, BCL2L2, CTGF, EGF	95.58
BIX-01294	DNA methyltransferase inhibitor, histone lyinse methyltransferase inhibitor, histone lysine methyltransferase inhibitor	EHMT1, EHMT2	95.41
rhodomyrtoxin-b	cytotoxic, DNA intercalator		94.76
AG-592	tyrosine kinase inhibitor		94.6
embelin	HCV inhibitor, XIAP inhibitor	XIAP	94.08
lasalocid	ionophore antibiotic		94
YM-155	survivin inhibitor, XIAP expression inhibitor	BIRC5	93.55
L-690488	nositol monophosphatase inhibitor	IMPA1	93.38
tyrphostin-AG-1478	EGFR inhibitor	EGFR, MAPK14	93.02
QW-BI-011	histone lyinse methyltransferase inhibitor	EHMT2	92.7
JTC-801	opioid receptor antagonist	OPRL1	92.21

Table S24. cMap_Compound_SC144_LN CaP_Down

Name	Description	Target	Median_score
benzo(a)pyrene	pro carcinogen that is metabolized to the DNA intercalating agent benzo(a)pyrene diol epoxide		-98.98
PPP2R5B	Serine/threonine phosphatases / Protein phosphatase 2, regulatory subunits, protein phosphatase 2, regulatory subunit B', beta		-96.23
TEX10	testis expressed 10		-88.57
troglitazone	PPAR receptor agonist, insulin sensitizer, CCK ligand expression inhibitor, EGR1 expression enhancer, glycogen synthase kinase stimulant	PPARG, ACSL4, AKR1B1, CCL2, CYP3A4, ESRRA, ESRRG, INS, SERPINE1, SLC29A1, TRPM3	-87.06
GNB2	WD repeat domain containing, guanine nucleotide binding protein (G protein), beta polypeptide 2		-87.02
TRAF4	RING-type (C3HC4) zinc fingers, TNF receptor-associated factor 4		-86.87
CASP4	Caspases, caspase 4, apoptosis-related cysteine peptidase		-86.4
PP-2	src inhibitor	SRC, LCK, ABL1, LYN, RIPK2	-85.03
ZG-10	JNK inhibitor	MAPK8	-84.81
PRKCQ	Delta subfamily, protein kinase C, theta		-84.67
bisindolylmaleimide	CDK inhibitor, PKC inhibitor, leucine rich repeat kinase inhibitor	CCND1, CDK4, LRRK2, PDPK1, PIM1, PRKCA, PRKCB, PRKCI, PRKCZ	-84.57
diethylstilbestrol	estrogen receptor agonist, chloride channel blocker	ESR1, ESR2, ESRRG, ESRRB	-84.49
CPD	Carboxypeptidase A, carboxypeptidase D		-84.15
INHBE	inhibin, beta E		-83.83
SOS1	Rho guanine nucleotide exchange factors, son of sevenless homolog 1 (Drosophila)		-83.65
TWS-119	glycogen synthase kinase inhibitor	GSK3B, JUN, MYC	-82.72
PHTF2	putative homeodomain transcription factor 2		-82.67
ALAS1	aminolevulinate, delta-, synthase 1		-82.16
MYO10	Pleckstrin homology (PH) domain containing, myosin X		-82.02
FXYD2	Ion transport regulator, FXYD domain containing ion transport regulator 2		-81.81
SGCB	sarcoglycan, beta (43kDa dystrophin-associated glycoprotein)		-81.79
dephostatin	tyrosine phosphatase inhibitor	PTPN1, PTPN6	-81.76
bicuculline	GABA receptor antagonist	GABRA1, GABRA2, GABRA3, GABRA4, GABRA5, GABRA6, KCNN1	-81.37

MLEC	malectin	-81.36
HERPUD1	homocysteine-inducible, endoplasmic reticulum stress-inducible, ubiquitin-like domain member 1	-80.65
